# *Salvia miltiorrhiza* ultrafine powder improves ileal barrier function and health of hens during late laying period via TLR4/NF-κB-mediated macrophage polarization

**DOI:** 10.3389/fimmu.2026.1934610

**Published:** 2026-08-20

**Authors:** Chengwen Meng, Md Abul Kalam Azad, Wenchao Lin, Jue Gui, Yadong Cui, Wei Lan, Xiangfeng Kong

**Affiliations:** 1 Hunan Provincial Key Laboratory of Animal Nutritional Physiology and Metabolic Process, National Engineering Laboratory for Pollution Control and Waste Utilization in Livestock and Poultry Production, Institute of Subtropical Agriculture, Chinese Academy of Sciences, Changsha, Hunan, China; 2 College of Advanced Agricultural Sciences, University of Chinese Academy of Sciences, Beijing, China; 3 School of Biology and Food Engineering, Fuyang Normal University, Fuyang, China

**Keywords:** gut microbiota, laying hens, macrophage polarization, network pharmacology, *Salvia miltiorrhiza* ultrafine powder, tanshinone IIA, TLR4/NF-κB pathway

## Abstract

**Background:**

Declining laying performance and egg quality during the late laying period are closely associated with intestinal barrier dysfunction, chronic inflammation, and gut microbiota dysbiosis. In our previous study, dietary supplementation with 15 g/kg *Salvia miltiorrhiza* ultrafine powder (SMUP) exhibited the greatest improvement in laying performance and egg quality of hens during late laying period. However, the underlying mechanisms remain unclear. Therefore, the present study further investigate the effects of dietary SMUP on ileal morphology, barrier function, immune response, and gut microbiota, combined with *in silico* analyses to elucidate the potential molecular mechanisms.

**Methods:**

A two-group feeding trial with late-laying hens was conducted to evaluate ileal histomorphology, tight junction protein expression, plasma and ileal inflammatory cytokine levels, and ileal microbial community composition through 16S rRNA sequencing. Transcriptomic data from LPS-stimulated chicken HD11 macrophages (GSE278197) were analyzed by differential expression and weighted gene co-expression network analysis (WGCNA) to identify macrophage polarization hub genes. Network pharmacology, molecular docking and 100 ns molecular dynamics simulations were sequentially performed to identify the key bioactive compound of SMUP and its target protein related to intestinal immune regulation.

**Results:**

Dietary SMUP (15 g/kg) reduced ileal crypt depth (CD), increased villus height/CD ratio, upregulated tight junction proteins (*claudin-1*, *occludin*, and *ZO-1*), and inhibited the TLR4/NF-κB signaling pathway by downregulating *TLR4* and *NF-κB p65* expressions (*P* < 0.05). Additionally, SMUP downregulated pro-inflammatory cytokines (*IL-1β*, *IL-6*, *TNF-α*, *IFN-γ*, and *iNOS*), elevated anti-inflammatory cytokines (*IL-4* and *IL-10*) expressions in the ileum, and increased plasma IL-10 level (*P* < 0.05). Dietary SMUP also modulated gut microbiota by reducing the Tenericutes abundance and enriching predicted ansamycin biosynthesis. Furthermore, network pharmacology, molecular docking, and molecular dynamics simulations identified tanshinone IIA as a core compound directly targeting NFKBIA (encoding IκBα), suggesting that SMUP stabilizes IκBα, thereby blocking its phosphorylation-dependent degradation and subsequent NF-κB nuclear translocation. Through inhibition of the TLR4/NF-κB pathway, SMUP promoted the polarization of intestinal macrophages from the pro-inflammatory M1 toward the anti-inflammatory M2 phenotype, which was consistent with the reciprocal changes in M1/M2-related gene expression.

**Conclusion:**

Dietary SMUP improves intestinal health of late-laying hens by strengthening the intestinal barrier, alleviating inflammation (via inhibiting the TLR4/NF-κB pathway and promoting M2-type macrophage polarization), and modulating the gut microbiota. These improvements in intestinal health supported SMUP as a promising natural plant-derived feed additive for hens during late laying period.

## Graphical Abstract

**Figure:**
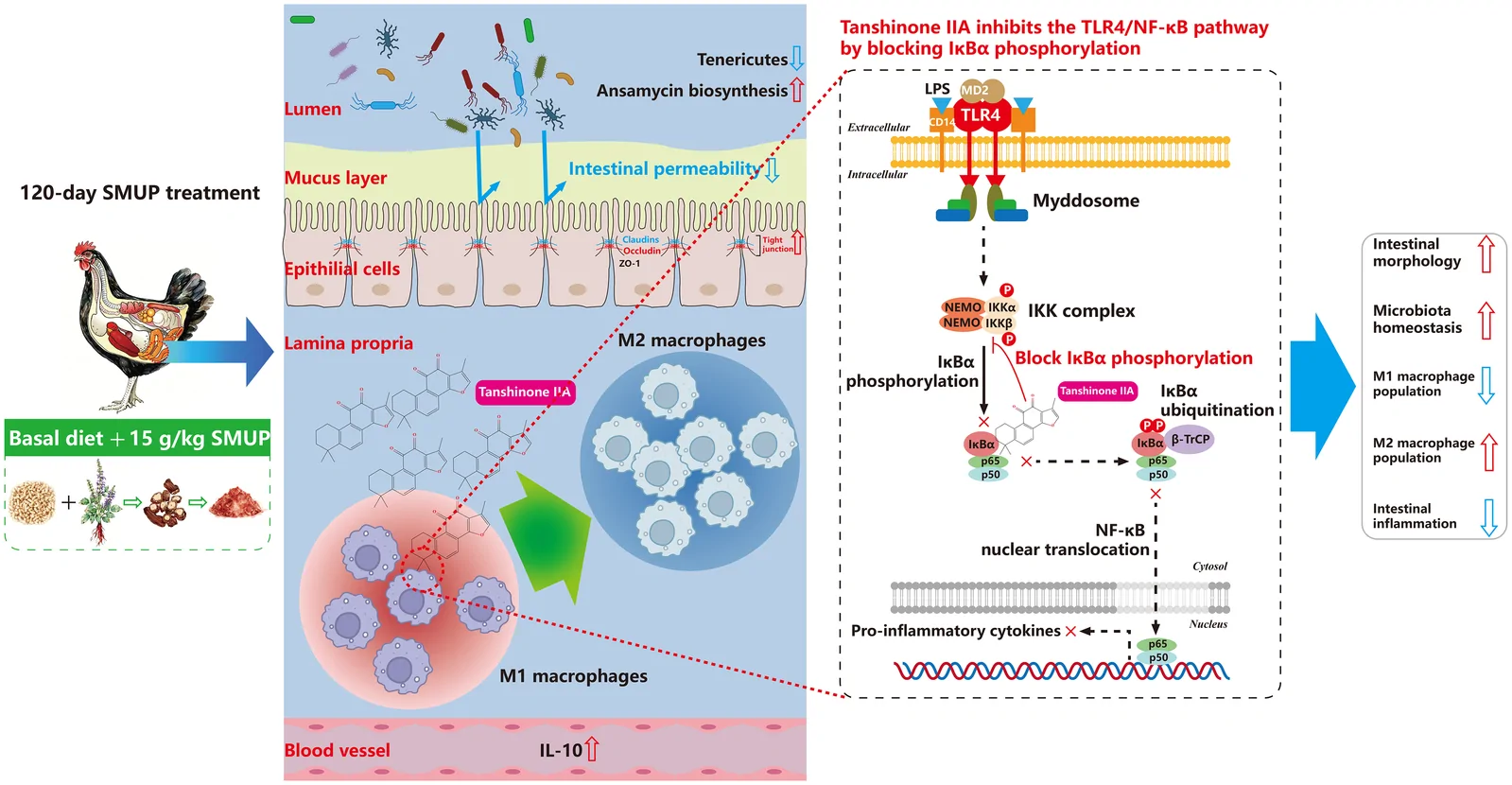

## 1 Introduction

The gastrointestinal tract of vertebrates is colonized by a vast community of bacteria commonly called gut microbiota (1). A stable and diverse intestinal microbiota plays a vital role in maintaining the normal digestive, absorptive, and immune functions of the host, thereby influencing the overall performance of animals (2). Once the homeostasis between intestinal microbiota and barriers is disrupted, exogenous harmful substances can penetrate the intestinal mucosa and enter the tissues and blood circulation, causing inflammation and diseases (3). Compromised intestinal health—including impaired morphology (4), disrupted mechanical barrier (5), chronic inflammation (6), and gut microbiota dysbiosis (7)—is recognized as one of the major contributors to declining laying performance and egg quality during the late laying period.

*Salvia miltiorrhiza* (SM) is a well-known perennial traditional Chinese herb, which belongs to the mint family Lamiaceae. It was first described in *Shennong’s Classic of Materia Medica*, a classic text compiled during the *Eastern Han* Dynasty of China (25–220 C.E.), and has been officially included in the *Pharmacopoeia of the People’s Republic of China* (8). There are two main bioactive compound categories in SM, namely liposoluble diterpenoid quinones (e.g., tanshinone IIA and cryptotanshinone) and water-soluble phenolic acids (e.g., salvianolic acid A and salvianolic acid B). These compounds, particularly tanshinones, have been reported to improve intestinal morphology, strengthen intestinal barrier function, and alleviate intestinal inflammation. For example, tanshinone IIA dose-dependently reduced intestinal permeability, bacterial translocation rate, and malondialdehyde, IL-1β, and TNF-α levels in both serum and ileum of septic mice, while increasing the protein abundances of claudin-1, occludin, and ZO-1 in the ileum (9). Similarly, cryptotanshinone has been demonstrated to markedly inhibit DSS-induced ulcerative colitis in mice, suppressing inflammatory responses by reducing STAT3 phosphorylation level in colonic tissue and the proportion of Th17 cells in the spleen and mesenteric lymph nodes, while also increasing colon length and lowering histological scores in chronic colitis (10). However, most of these studies focused on mammalian models, and reports on the effects of SM or its extracts on intestinal health and microbiota of late-laying hens are still very limited. In our previous study (11), dietary supplementation with 15 g/kg SM ultrafine powder (SMUP) significantly reduced the feed conversion ratio, enhanced eggshell strength, promoted follicular development, and increased laying rate over a 120-day trial period. Based on the above findings, we hypothesized that dietary SMUP can strengthen the intestinal barrier, alleviate chronic intestinal inflammation, and improve gut microbiota composition, thereby enhancing laying performance and egg quality in late-laying hens.

Traditional Chinese herbs contain multiple bioactive compounds and exert biological effects through multi-target mechanisms. However, it is difficult to clarify their precise mechanisms of action using conventional research methods (12). Network pharmacology allows the screening of key bioactive compounds and their corresponding targets through the construction of herb–compound–target and protein–protein interaction (PPI) networks (13). As a computer simulation technique, molecular docking predicts binding sites and energies between ligands (key bioactive compounds) and receptors (key targets), thereby validating analytical results from network pharmacology (14). Notably, to improve computational speed, molecular docking uses a semi-flexible docking mode, with small molecule ligands remaining flexible and adopting variable conformations, while protein receptors are set in a rigid state (15). Nevertheless, receptor proteins also exhibit structural flexibility during ligand binding under *in vivo* physiological conditions (16). In addition, molecular docking mainly predicts a single static conformation with the lowest binding energy at a specific time point, whereas the binding process between receptors and ligands is continuous and dynamic, involving numerous conformational transitions *in vivo* (17). Therefore, molecular docking cannot fully reflect the actual binding characteristics of ligand–receptor interactions. Unlike molecular docking, molecular dynamics simulations are performed under physiologically realistic temperature, pressure, and solvent conditions. This approach accounts for receptor protein flexibility and performs continuous dynamic calculations over a specified period, thereby better reflecting the authentic binding behaviors between receptors and ligands (18). By integrating these three computational approaches—network pharmacology for target screening, molecular docking for binding mode prediction, and molecular dynamics simulations for binding stability validation—we aimed to establish a reliable *in silico* workflow that compensates for the limitations of each individual method and provides a robust mechanistic interpretation of our *in vivo* observations. Combining *in vivo* animal trials with multi-layered computational analyses can help link phenotypic changes to underlying molecular targets. Therefore, the present study evaluated ileal morphology, microbial diversity and function, and barrier- and immune-related factors in late-laying hens, and further integrated network pharmacology, molecular docking, and molecular dynamics simulations to systematically elucidate the underlying molecular mechanisms of dietary SMUP in regulating intestinal function and microbiota homeostasis.

## 2 Materials and methods

### 2.1 Birds, experimental design, and feeding management

Our previous study found that dietary supplementation with 15 g/kg of SMUP resulted in an improvement in laying performance and egg quality (11). Accordingly, the present study further determined other indices in the CON and SMUP15 groups, including plasma immune parameters, ileal immune-related gene expression, ileal microbial community composition, and ileal histomorphology, to investigate the potential mechanisms underlying the enhancement of laying performance and egg quality in laying hens during the late laying period by SMUP.

A total of 144 healthy Xinyang black-feather laying hens at 307 days of age with normal laying performance were randomly divided into two groups, with eight replicates per group and nine hens per replicate. After a 7-day adaptation, birds in the control (CON) group received a corn-soybean based basal diet, while birds in the 15 g/kg SMUP supplementation (SMUP15) group received a basal diet supplemented with 15 g/kg SMUP. The diet was formulated to meet the Nutrient Requirements of Poultry recommended by National Research Council (1994; **Supplementary Table 1**). The feeding experiment lasted 120 days and was carried out in a large specialized commercial layer farm (capacity of 60,000 hens) of Huisiyuan Agriculture and Animal Husbandry Co., Ltd., Fuyang, China. The dried roots of SM were purchased from Kangxing Pharmaceutical Co., Ltd., Zhengzhou, China. To prepare SMUP, the dried roots were pulverized and passed through a 300-mesh sieve, with > 95% of particle sizes < 48 μm and < 1% exceeding 75 μm. The SMUP was mixed uniformly with the basal diet before feeding. The hens were housed in wire cages with three hens per cage and uniformly kept in the second layer of the four-tier ladder-type cages in an enclosed layer house to ensure consistent lighting and facilitate daily feeding and management. All hens had free access to clean water. The temperature in the layer house was maintained below 28 °C using a cooling pad system combined with longitudinal negative-pressure ventilation. The lighting regimen was set at 16 h light and 8 h dark per day, provided by LED lamps with an intensity of 15–20 lux.

### 2.2 Sample collection

At the end of the trial, one bird was randomly selected from each replicate (total eight birds for each group) for sampling. After fasting 12 h, blood samples (5 mL) were drawn from the wing vein into heparin sodium tubes, and centrifuged for 10 min at 3000 × *g* and 4°C to collect plasma. Plasma samples were immediately stored at −20 °C for the determination of immune-related parameters. The hens were euthanized by cutting the jugular vein after the collection of blood samples. Two 1-cm sections of the ileum (proximal of the ileocecal junction) were quickly excised and gently flushed with saline, then one section was fixed in 4% paraformaldehyde solution for histomorphology analysis, and the other was snap-frozen in liquid nitrogen and then stored at −80 °C until gene expression analysis. Moreover, the ileal luminal contents were collected in 1.5 mL tubes and stored at −80 °C for microbiota DNA extraction.

### 2.3 Determination of plasma immune parameters

Plasma immunoglobulin G (IgG), interleukin (IL)-1β, IL-4, IL-6, IL-10, IL-13, interferon-γ (IFN-γ), and tumor necrosis factor α (TNF-α) were determined by using the commercially available ELISA kits (Meimian Industrial Co., Ltd., Yancheng, China) on a TECAN Inffnite M200 PRO microplate reader (Mannedorf, Switzerland), according to the manufacturer’s instructions.

### 2.4 Analysis of ileal histomorphology

Fixed ileal tissues were trimmed, cassetted, and rinsed overnight in running tap water. Samples were dehydrated with graded ethanol, cleared in xylene–ethanol mixture and two sequential pure xylene baths, and then infiltrated at controlled temperatures with xylene–paraffin blend and two grades of molten paraffin. Tissues were embedded in molten paraffin, and solidified wax blocks were trimmed and sectioned into 4–6 μm slices with a microtome. Sections were floated on ~40 °C warm water, mounted on slides, and dried at 45 °C for 12 h. Slides were dewaxed in xylene, rehydrated via descending graded ethanol, and washed in distilled water. Specimens then underwent H&E staining.

Staining quality and intestinal morphology were first examined under a conventional light microscope to ensure normality. Qualified slides were scanned into mrxs format using a Pannoramic SCAN II digital slide scanner (3DHistech, Budapest, Hungary). The mrxs files were opened and analyzed with CaseViewer 2.0 software (3DHistech, Budapest, Hungary). Six of the longest intact villi were selected from each section for measurement of villus height (VH) and corresponding crypt depth (CD), and the VH to CD ratio (VCR) was calculated.

### 2.5 RNA extraction, reverse transcription PCR, and quantitative real-time PCR

The total RNA was extracted from the ileal tissues using TRIzol reagent (Accurate Biotechnology Co., Ltd., Changsha, China) according to the manufacturer’s instructions. The purity and concentration of extracted RNA were determined using a NanoDrop ND-2000 UV spectrophotometer (Thermo Fisher Scientifific, Waltham, MA, USA). Afterward, 1 mg of total RNA was reverse transcribed into cDNA using an Evo M-MLV RT Kit (Accurate Biotechnology Co., Ltd., Changsha, China) according to the manufacturer’s protocol. The expression of targeted genes (listed in **Supplementary Table 2**) was quantified using the SYBR Green Premix *Pro Taq* HS qPCR Kit (Accurate Biotechnology Co., Ltd., Changsha, China) on a LightCycler^®^ 480 II Real-time PCR System (Roche, Basel, Switzerland). For quantitative real-time PCR, the cDNA was diluted 20-fold with DEPC-treated water prior to amplification. The reaction mixture (10 μL total volume) consisted of 4.2 μL of diluted cDNA, 5.0 μL of SYBR Green Premix *Pro Taq* HS qPCR mix, 0.4 μL of forward primer (10 μM), and 0.4 μL of reverse primer (10 μM). The amplification program was set as follows: initial denaturing at 95 °C for 30 s, followed by 40 cycles of 95 °C for 5 s and 60 °C for 30 s, with a final melting curve analysis performed from 60 °C to 95 °C to verify the specificity of the amplified products. The 2 ^-ΔΔCt^ method was used to calculate the relative mRNA expression level of each gene (19), with housekeeping gene β-actin as the internal reference.

### 2.6 Genomic DNA extraction, target region PCR amplification, library construction, and 16S rDNA sequencing of ileal microbiota

Genomic bacterial DNA of ileal contents was extracted using E.Z.N.A.^®^ Stool DNA Extraction Kit (Omega Bio-Tek, Norcross, USA). The purity and concentration of extracted DNA were determined using a NanoDrop ND-2000 UV spectrophotometer (Thermo Fisher Scientifific, Waltham, MA, USA), and DNA integrity was checked on 1.2% agarose gel electrophoresis. The V3−V4 hypervariable region (~468 bp) of 16S rDNA genes amplified with universal primers 338F (5’-ACTCCTACGGGAGGCAGCA-3’) and 806R (5’-GGACTACHVGGGTWTCTAAT-3’). The PCR products were detected on a 2% agarose electrophoretic gel, and the target fragment was recovered with AxyPrep™ DNA gel Extraction kit (Axygen Scientific, Inc., Union City, CA, USA). The recovered PCR products were quantified using the Quant-iT PicoGreen dsDNA Assay Kit (Invitrogen Corporation, Carlsbad, CA, USA) on a FLx800™ Microplate reader (BioTek Instruments, Inc., Winooski, VT, USA). Afterwards, each sample was mixed in a proportion based on the quantitative result and the sequencing quality requirement. These mixed samples were used to construct the sequencing library using the TruSeq Nano DNA LT Library Prep Kit (Illumina, Inc., San Diego, CA, USA). Constructed libraries were detected by the Bioanalyzer 2100 (Angilent, Santa Clara, CA, USA), and quantified using the QuantiFluor™-ST (Promega, Madison, WI, USA) with the Quant-iT PicoGreen dsDNA Assay Kit (Invitrogen Corporation, Carlsbad, USA). Finally, the qualified libraries were paired-end sequenced (2 × 250 bp) on an Illumina Novaseq 6000 platform (Illumina, Inc., San Diego, CA, USA) at Shanghai Personal Biotechnology Co., Ltd. (Shanghai, China) by using the NovaSeq 6000 SP Reagent Kit (Illumina, Inc., San Diego, CA, USA).

### 2.7 Bioinformatic analysis of 16S rDNA sequencing data of ileal microbiota

The raw data obtained from 16S rDNA sequencing were demultiplexed and quality-filtered using Quantitative Insights into Microbial Ecology 2 (QIIME2 2019.4, https://Qiime2.org) software. The R package “Division Amplicon Denoising Algorithms 2 (DADA2)” was used for the remainder of the processing. Briefly, the demultiplexed “fastq” files for each sample were filtered, trimmed, and dereplicated to estimate the error rates. Forward/reverse reads were merged, and chimeras were removed from the whole set to generate the amplicon sequence variant (ASV) table. Singleton ASVs were discarded. Each sample was randomly subsampled to the 95% of the lowest sample sequence size. A Naïve Bayes taxonomy classifier was pulled on the Greengenes reference database (https://greengenes.lbl.gov/Download/). Chao1, Observed_species, Shannon, and Simpson indexes were used to measure alpha-diversity. Beta diversity analysis with principal coordinates analysis (PCoA) was based on the Bray-Curtis distance. Taxa abundances at the phylum (top 10) and genus (top 15) levels, as well as the ratio of *Firmicutes* to *Proteobacteria*, were compared between the two groups using the Mann-Whitney U test. Linear discriminant analysis (LDA) coupled with effect size measurement (LEfSe) was used to identify the differences in microbial composition between the two groups. The biological functions of ileal microbiota were predicted using the Phylogenetic Investigation of Communities by Reconstruction of Unobserved States (PICRUSt2) software, based on the Kyoto Encyclopedia of Genes and Genomes (KEGG) database from 16S rDNA sequencing data, and grouped by KEGG hierarchical level 3. The Statistical Analysis of Metagenomic Profiles (STAMP) software was used to analyze and visualize the predicted metagenomic functions. For STAMP, Welch’s t tests (two-tailed) were used for comparisons between groups, and the confidence interval was set at 95%. Pearson correlation analysis was performed on the expression levels of immune-related genes in ileal tissues. Mantel tests were separately carried out between the expression matrix of immune-related genes and either the abundance matrix of microbes with an LDA score > 3 or the matrix of significantly improved economic indicators. The data for these improved economic indicators were obtained from our previous study (11). Visualization was subsequently implemented using the linkET package in R.

### 2.8 Acquisition and analysis of GEO dataset

We adopted the raw count matrix from a high-throughput transcriptomic sequencing dataset (GSE278197) in the Gene Expression Omnibus (GEO) database (https://www.ncbi.nlm.nih.gov/geo/). The dataset was based on the chicken macrophage-like HD11 cell model, and included four treatment groups with three biological replicates per group. Among them, the control group (treated with PBS) and LPS group (treated with lipopolysaccharide derived from *Escherichia coli* serotype O55:B5) were selected (20). We used data from these two groups to perform differential expression analysis and weighted gene co-expression network analysis (WGCNA). Genes satisfying both |log_2_FC| > 1 and adjusted *P*-value (Benjamini–Hochberg correction) were defined as differentially expressed genes (DEGs). Gradient volcano plots and hierarchical clustering heatmaps for differential expression analysis were generated in R software.

We conducted WGCNA and its visualization using the WGCNA package in R (21). First, the raw count matrix was read in, and genes with low expression level were filtered, retaining genes with a count > 10 in at least three samples to avoid noise. Subsequently, the raw count matrix was transformed using the variance stabilizing transformation (VST) from DESeq2. Afterward, a sample clustering tree was constructed to check sample clustering and ensure reasonable grouping. Pearson’s correlation coefficient between each pair of genes was calculated to build a co-expression correlation matrix, and the absolute values of the correlation coefficients were raised to the power β (β ≥ 1) to construct an adjacency matrix. The optimal soft threshold was determined as the lowest β value that achieved a scale-free topology fitting index (R²) > 0.85 and stabilized the mean connectivity. Based on the adjacency matrix constructed with the optimal soft threshold, the topological overlap matrix (TOM) was calculated, and the dissimilarity matrix (1−TOM) was further generated. Hierarchical clustering was then performed on the dissimilarity matrix to cluster genes with similar expression patterns into modules, and the dynamic tree-cutting algorithm was applied to refine module division, ensuring more consistent gene expression within each module. Moreover, genes were grouped into multiple modules, and those within the same module were considered to be functionally related. A module-trait association analysis (Pearson correlation) was conducted to identify the module most relevant to LPS treatment, and genes in this module were considered as candidate genes for macrophage polarization. Finally, the intersection of DEGs and genes in the relevant module was identified as the hub genes associated with macrophage polarization in the GSE278197 dataset.

### 2.9 Network pharmacology analysis

Potential bioactive compounds in SM were identified using the Traditional Chinese Medicine Systems Pharmacology Database and Analysis Platform (TCMSP) (https://tcmsp-e.com/tcmspsearch.php). Compounds that met both oral bioavailability (OB) ≥ 30% and drug-likeness (DL) ≥ 0.18 were defined as potential bioactive compounds. In addition, according to the *Pharmacopoeia of the People’s Republic of China* (8) and several published articles (22–25), several compounds that did not meet OB ≥ 30% and/or DL ≥ 0.18 but were widely reported were also included in this study. The targets of the potential bioactive compounds were searched using five databases: TCMSP, SwissTargetPrediction (http://swisstargetprediction.ch/), ChEMBL (https://www.ebi.ac.uk/chembl/), PharmMapper (https://www.lilab-ecust.cn/pharmmapper/), and the HERB database (http://herb.ac.cn/). The final targets of the potential bioactive compounds (FTBCs) from SM were then obtained by merging and deduplicating the predicted targets from the five databases.

Targets were retrieved from the GeneCards database (https://www.genecards.org/) using the keywords “*Gallus gallus macrophage polarization*” and “*Chicken macrophage polarization*”, and the corresponding data were downloaded. These targets were then filtered by applying a threshold of twice the median relevance score. After filtering, the remaining targets were merged and deduplicated to obtain the known targets associated with chicken macrophage polarization from the database. Subsequently, the final targets related to chicken macrophage polarization (FTMPs) were identified by intersecting the database-derived targets with the hub genes obtained from the GSE278197 dataset. Finally, the overlap between the FTBCs and FTMPs was defined as the potential targets of bioactive compounds from SM that modulate the chicken macrophage polarization process (PTSMs).

PTSMs were imported into Cytoscape 3.10.0 to construct a SM–compound–target topological network, which was further analyzed for its network function. Compounds with higher degree values were considered potential key compounds. Afterwards, PTSMs were uploaded into the STRING database (https://cn.string-db.org/) with “*Organisms*” set to “*Gallus gallus*” to construct a PPI network. Both original images of the PPI network and the corresponding tsv files were retrieved. The tsv files were then imported into Cytoscape 3.10.0 for further topological network analysis and visualization. The Centiscape 2.2 plugin was used to calculate the degree, closeness, and betweenness of nodes in the PPI network, and potential key targets were screened based on their degree values. Subsequently, the PTSMs were submitted to the DAVID database (https://davidbioinformatics.nih.gov/) with “Select species” set to “*Gallus gallus*” to perform Gene Ontology (GO) and Kyoto Encyclopedia of Genes and Genomes (KEGG) pathway enrichment analysis. The GO systematically classifies genes into three structured ontologies: biological process (BP), cellular component (CC), and molecular function (MF). The enrichment results were visualized using the online platform Bioinformatics (www.bioinformatics.com.cn). To identify candidate core targets (CCTs), the intersection of targets enriched in the KEGG pathway with the lowest *P*-value (core pathway) among PTSMs and the top 10 targets with the highest degree values in the PPI network constructed from PTSMs. The distribution of these CCTs in the core pathway was observed, and the target located upstream of the pathway that exerts critical regulatory functions was defined as the core target.

### 2.10 Molecular docking analysis

Molecular docking analysis was performed using the core target proteins as receptors, while the compounds with a degree value > 5 in the SM–compound–target network served as ligands. In this study, NFKBIA was used as the receptor protein. First of all, experimentally determined 3D structure of human NFKBIA (PDB ID: 1NFI) were downloaded from the PDB database with a resolution of 2.70 Å. After selecting appropriate structures, they were saved in PDB format, imported into PyMOL 2.6 software to remove water molecules and original ligands, and then re-saved as PDB files for further use. Since the PDB database (https://www.rcsb.org/) contains very few experimentally measured protein structures for chicken (*Gallus gallus*), AlphaFold 3 was used to predict the 3D structures of the chicken core targets for subsequent molecular docking analyses. Briefly, the amino acid sequences of corresponding proteins were retrieved from the UniProt database (https://www.uniprot.org/), uploaded to AlphaFold 3 for structure prediction, and the resulting atomic-level 3D structures were downloaded and stored in PDB format. Subsequently, the mol2 format structures of the bioactive compounds were downloaded from the TCMSP database. The pretreatment of receptors and ligands before molecular docking, as well as the parameter settings during the docking process, were similar to those described in our previous study (11). For the experimentally determined human NFKBIA structure (PDB ID: 1NFI), the grid box was centered at x_center = −5.239, y_center = 67.700, z_center = 74.260 with dimensions of x_size = 54, y_size = 54, z_size = 90, and a spacing of 0.431 Å. For the AlphaFold 3-predicted chicken NFKBIA structure, the grid box was centered at x_center = −3.533, y_center = 1.063, z_center = 5.884 with dimensions of x_size = 328, y_size = 292, z_size = 350, and a spacing of 0.375 Å. Molecular docking was performed using AutoDock Tools 1.5.6 with the Lamarckian genetic algorithm, and the number of docking runs was set to 10 for each ligand–receptor pair. Other parameters were set as default, including a population size of 150, a maximum number of 2,500,000 energy evaluations, and a maximum of 27,000 generations. After docking, the conformations with the lowest binding energy were selected, exported in pdbqt format, and then converted to PDB format using OpenBabel 3.1.1. The 3D and 2D visualizations were generated using PyMOL 2.6 and Discovery Studio 2019, respectively. Additionally, a heatmap of the molecular docking binding energies was plotted.

### 2.11 Molecular dynamics simulations

Molecular dynamics (MD) simulations were performed using GROMACS 2022.3 on the docking complex of the compound-ligand and chicken protein receptor pair that exhibited the lowest binding energy. Briefly, the docking complex in pdbqt format was converted to PDB format using Open Babel 3.1.1 and subsequently split using PyMOL 2.6. The protein receptor was saved in PDB format, and the ligand was saved in mol2 format. MD simulations were performed via a standardized pipeline. Ligands were hydrogenated and parameterized using AmberTools22 and Gaussian 16W for RESP charge calculation under the GAFF force field, with final files converted to GROMACS 2022.3-compatible formats via acpype. Receptor topology was generated using GROMACS pdb2gmx with the Amber99sb-ildn force field and TIP3P water model, with original protein hydrogens replaced by force field-standard residues. The processed ligand and receptor structures were merged to construct the complex system, and ligand topological parameters were integrated into the global system file. A cubic water box with a 1.2 nm buffer surrounding the complex was built and solvated, followed by ion neutralization with Na^+^ or Cl^−^ to eliminate residual system charge. The system was energy-minimized via the steepest descent algorithm for 50,000 steps until the maximum force decreased below 100 kJ·mol^−1^·nm^−1^. After minimization, 2 ns NVT equilibration (310 K, 2 fs timestep) was conducted using a V-rescale thermostat, followed by 2 ns NPT equilibration at 310 K and 1 bar with a Berendsen barostat. Ultimately, a 100 ns production MD simulation was executed under consistent isothermal-isobaric conditions.

Upon completion of the MD simulations, the raw trajectory was first preprocessed using the *gmx trjconv* tool. The parameters *-center*, *-pbc mol*, and *-ur compact* were applied sequentially to center the protein in the simulation box, correct molecular fragmentation caused by periodic boundary conditions, and compact the system. The trajectory was then fitted and aligned using the *-fit rot+trans* option to remove global rotational and translational motions of the protein, yielding a well-behaved trajectory suitable for subsequent analyses. Based on the preprocessed trajectory, the root-mean-square deviation (RMSD), root-mean-square fluctuation (RMSF), radius of gyration (Rg), and solvent-accessible surface area (SASA) were calculated. The number of hydrogen bonds between the protein and ligand was also monitored, and conformations of the protein–ligand complex at six time points (0, 20, 40, 60, 80, and 100 ns) were analyzed. To construct the free energy landscape, the *merge_rmsd_gyrate.py* script was first used to combine the RMSD and Rg data of the protein backbone. The *gmx sham* module in GROMACS was then employed to compute the Gibbs free energy landscape and generate the file *gibbs.xpm*. The *xpm2txt.py* script was used to convert *gibbs.xpm* into the text file *gibbs.txt*, and the *xpm2png_3D.py* script was applied to generate the 3D free energy landscape. To visually demonstrate the dynamic conformational changes of the complex, the *gmx trjconv* command was used to extract one frame every 1 ns from the 100 ns trajectory, yielding a total of 100 representative structures saved in PDB format. An animation of the MD simulation was subsequently generated using PyMOL 2.6. Further, the binding free energy of the protein–ligand complex was calculated using the molecular mechanics/generalized Born surface area (MM/GBSA) method. The binding free energy was:


ΔGbind=GRL−GR−GL

Where *GRL* is the free energy of the protein–ligand complex, *GR* is the free energy of the unbound protein, and *GL* is the free energy of the unbound ligand.

The binding free energy can be decomposed as:


ΔGbind=ΔH–TΔS

Where ΔH is the enthalpy change and *–TΔS* is the entropy change. *ΔH* can be further decomposed into *ΔGgas* (gas-phase molecular mechanics energy or intramolecular energy) and *ΔGsol* (solvation energy). *ΔGgas* consists of *ΔVDWAALS* (van der Waals energy) and *ΔEele* (electrostatic energy), while *ΔGsol* is composed of *ΔEGB* (polar solvation energy) and *ΔEsurf* (nonpolar solvation energy).

Because the calculation of *–TΔS* is computationally expensive and prone to systematic errors, it is often neglected in practical calculations. Consequently, only the enthalpy term is considered, yielding: *ΔTotal* = *ΔGbind* ≈ *ΔH* (26, 27).

### 2.12 Statistical analysis

Unless otherwise specified, all data in the present study were analyzed for significant differences using the independent-samples t-test in SPSS 26.0 software (SPSS Inc., Chicago, IL, USA). Before performing the independent-samples t-test, the normality of data distribution and homogeneity of variances were assessed using the Shapiro–Wilk test and Levene’s test, respectively, and the t-test was applied when both assumptions were satisfied. Data are expressed as means with their standard error of the mean (SEM). *P*-values < 0.05 were considered statistically significant.

## 3 Results

### 3.1 Effects of dietary SMUP on plasma immune parameters of laying hens

The effects of dietary SMUP on plasma immune parameters of laying hens during the late laying period are presented in **Table 1**. The plasma IL-10 concentration was increased (*P* < 0.001) in the SMUP15 group compared with the CON group. However, there were no significant differences in other plasma immune parameters between the two groups (*P* > 0.05).

**Table 1:** Effects of supplementation with *Salvia miltiorrhiza* ultrafine powder (SMUP) on plasma immune parameters of hens during late laying period.

Items	CON group	SMUP15 group	*P*-values
IgG (μg/mL)	1794.74 ± 91.88	1914.43 ± 68.01	0.332
IL-1β (pg/mL)	426.13 ± 17.48	521.47 ± 45.66	0.061
IL-4 (pg/mL)	136.32 ± 3.93	126.31 ± 3.16	0.074
IL-6 (pg/mL)	267.86 ± 7.54	288.20 ± 33.90	0.211
IL-10 (pg/mL)	65.47 ± 1.56	78.68 ± 2.08^**^	< 0.001
IL-13 (pg/mL)	651.24 ± 30.67	671.09 ± 38.83	0.695
IFN-γ (pg/mL)	238.40 ± 1.49	241.99 ± 3.26	0.335
TNF-α (pg/mL)	3772.80 ± 321.91	3519 ± 632.89	0.707

Data are expressed as means ± SEM (*n* = 8). ^**^P < 0.01. CON group, fed basal diet; SMUP15 group, fed basal diet supplemented with 15 g/kg SMUP; IgG, immunoglobulin G; IL, interleukin; IFN-γ, interferon-γ; TNF-α, tumor necrosis factor-α

### 3.2 Effects of dietary SMUP on ileal histomorphology of laying hens

**Figure 1:**
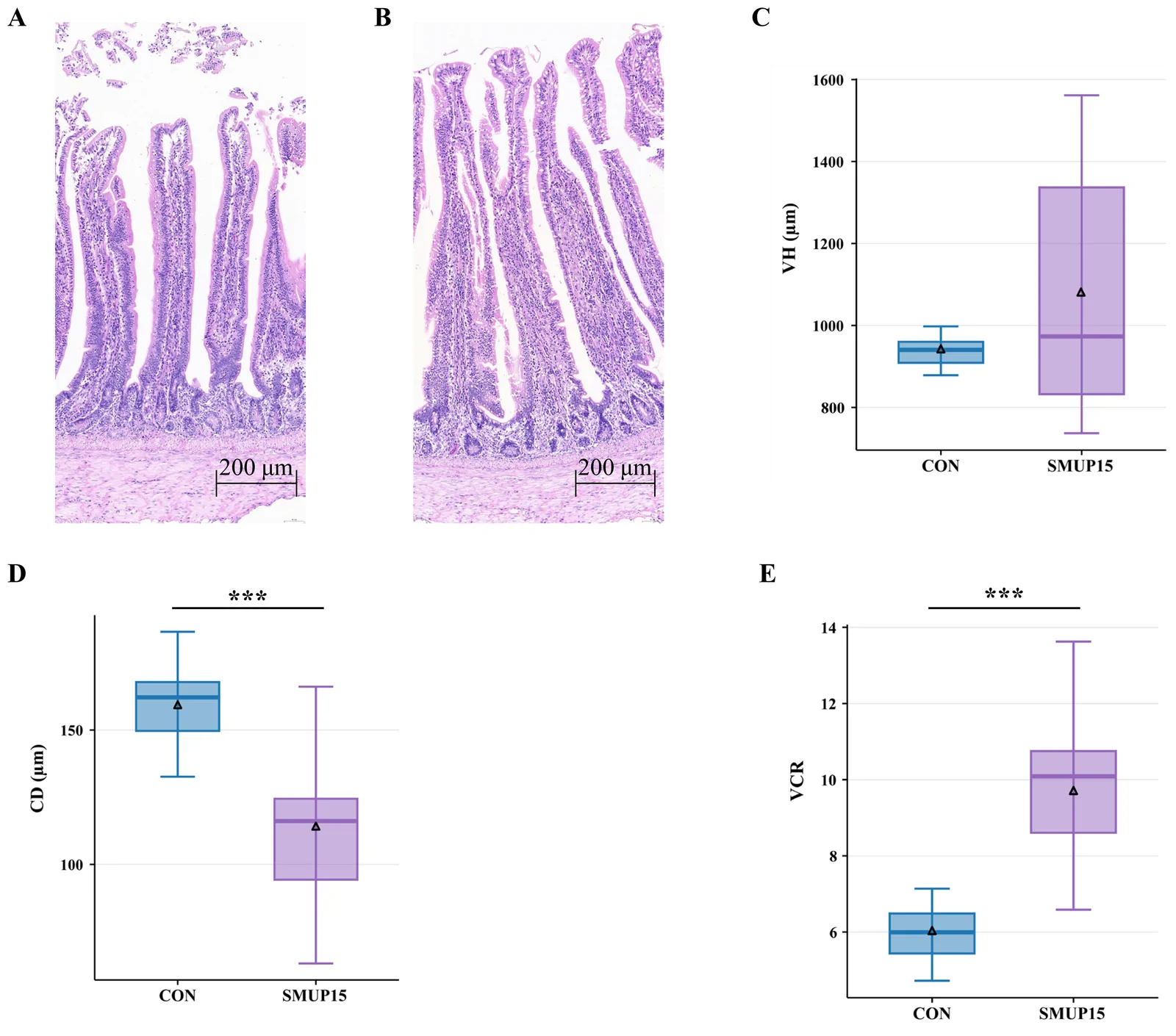
Effects of *Salvia miltiorrhiza* ultrafine powder (SMUP) on the ileal morphology (H&E staining). **(A)** Representative image in the CON group; **(B)** Representative image in the SMUP15 group; **(C)** Villus height (VH); **(D)** Crypt depth (CD); **(E)** Ratio of VH to CD (VCR). Scale bar is 200 μm. ^***^ *P* < 0.001. CON group, fed basal diet; SMUP15 group, fed basal diet supplemented with 15 g/kg SMUP.

As shown in **Figures 1A, B**, representative images of ileum morphology in the CON and SMUP15 groups are presented, respectively. As shown in **Figures 1C–E**, the CD was lower, and the VCR was higher in the SMUP15 group than in the CON group (*P* < 0.001), while there was no significant difference in VH between the two groups (*P* > 0.05).

### 3.3 Effects of dietary SMUP on the immune-related gene expression in the ileum of laying hens

**Figure 2:**
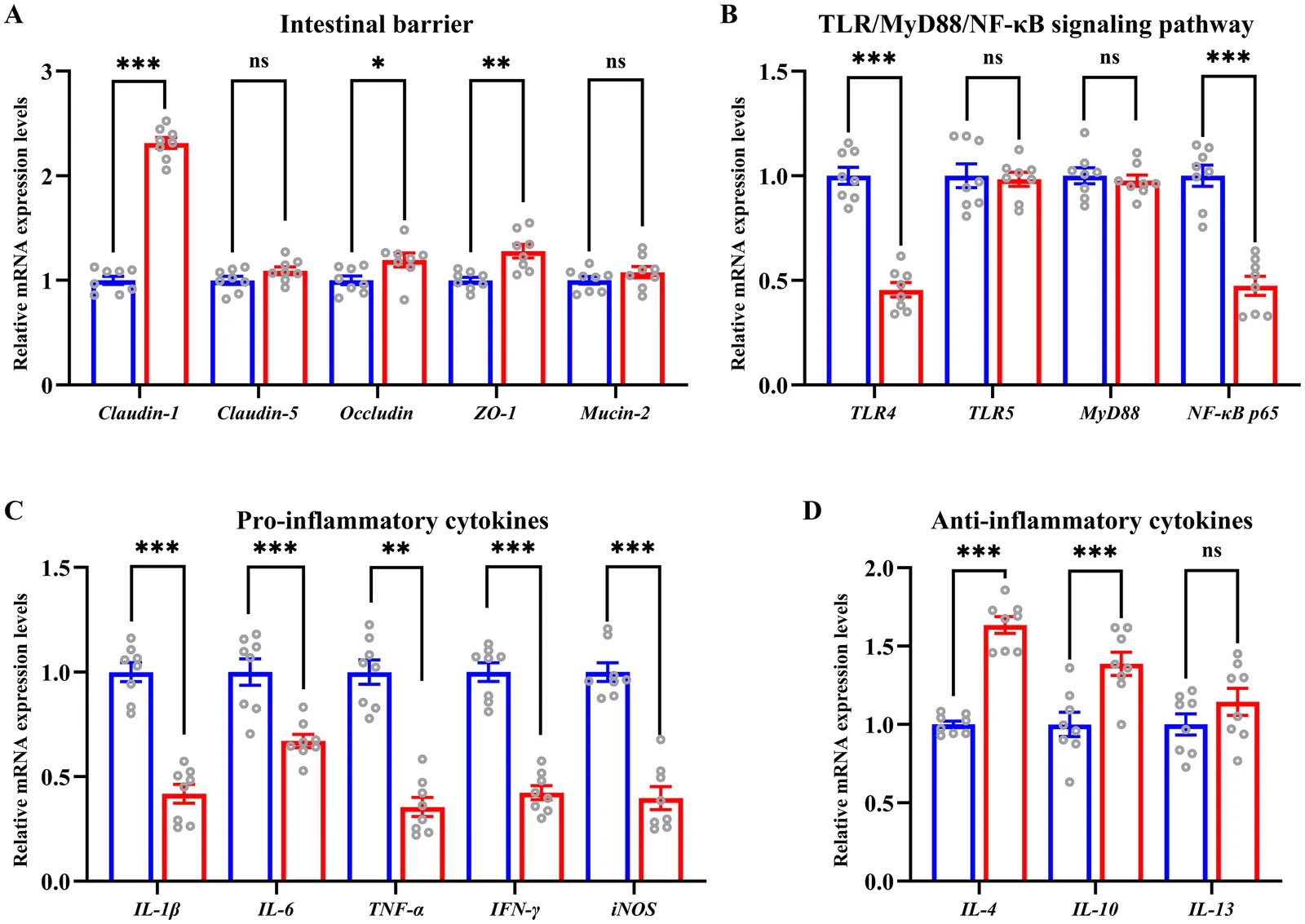
Effects of *Salvia miltiorrhiza* ultrafine powder (SMUP) on barrier and inflammation related gene expression in laying hens during the late laying period. **(A)** Genes related to ileal barrier function. **(B)** Genes related to TLR/MyD88/NF-κB signaling pathway. **(C)** Genes related to pro-inflammatory cytokines. **(D)** Genes related to anti-inflammatory cytokines. Data are expressed as means ± standard error of the mean (SEM). ^*^P < 0.05; ^**^P < 0.01; ^***^P < 0.001; ns, not significant. CON group, fed basal diet; SMUP15 group, fed basal diet supplemented with 15 g/kg SMUP. *ZO-1*, zonula occludens 1; *MUC2*, mucin-2; *TLR*, toll-like receptor; *MyD88*, myeloid differentiation factor 88; *NF-κB*, nuclear factor kappa B; *IL*, interleukin; *IFN-γ*, interferon-γ; *TNF-α*, tumor necrosis factor-α; *iNOS*, inducible nitric oxide synthase.

The effects of dietary SMUP on the relative mRNA expression of genes related to ileal barrier function and inflammation in laying hens during the late laying period are shown in **Figure 2**. The expression levels of *claudin-1* (*P* < 0.001), *occludin* (*P* < 0.05), and zonula occludens 1 (*ZO-1*) (*P* < 0.01) were upregulated (**Figure 2A**), while toll-like receptor 4 (*TLR4*) and nuclear factor kappa B p65 (*NF-κB p65*) were downregulated (*P* < 0.001) in the SMUP15 group compared with the CON group (**Figure 2B**). The expression levels of pro-inflammatory cytokines, including *IL-1β*, *IL-6*, *TNF-α*, *IFN-γ*, and inducible nitric oxide synthase (*iNOS*), were downregulated (*P* < 0.01; **Figure 2C**), while *IL-4* and *IL-10* were upregulated (*P* < 0.001; **Figure 2D**) in the SMUP15 group compared with the CON group.

### 3.4 Effects of dietary SMUP on ileal microbiota of laying hens

**Figure 3:**
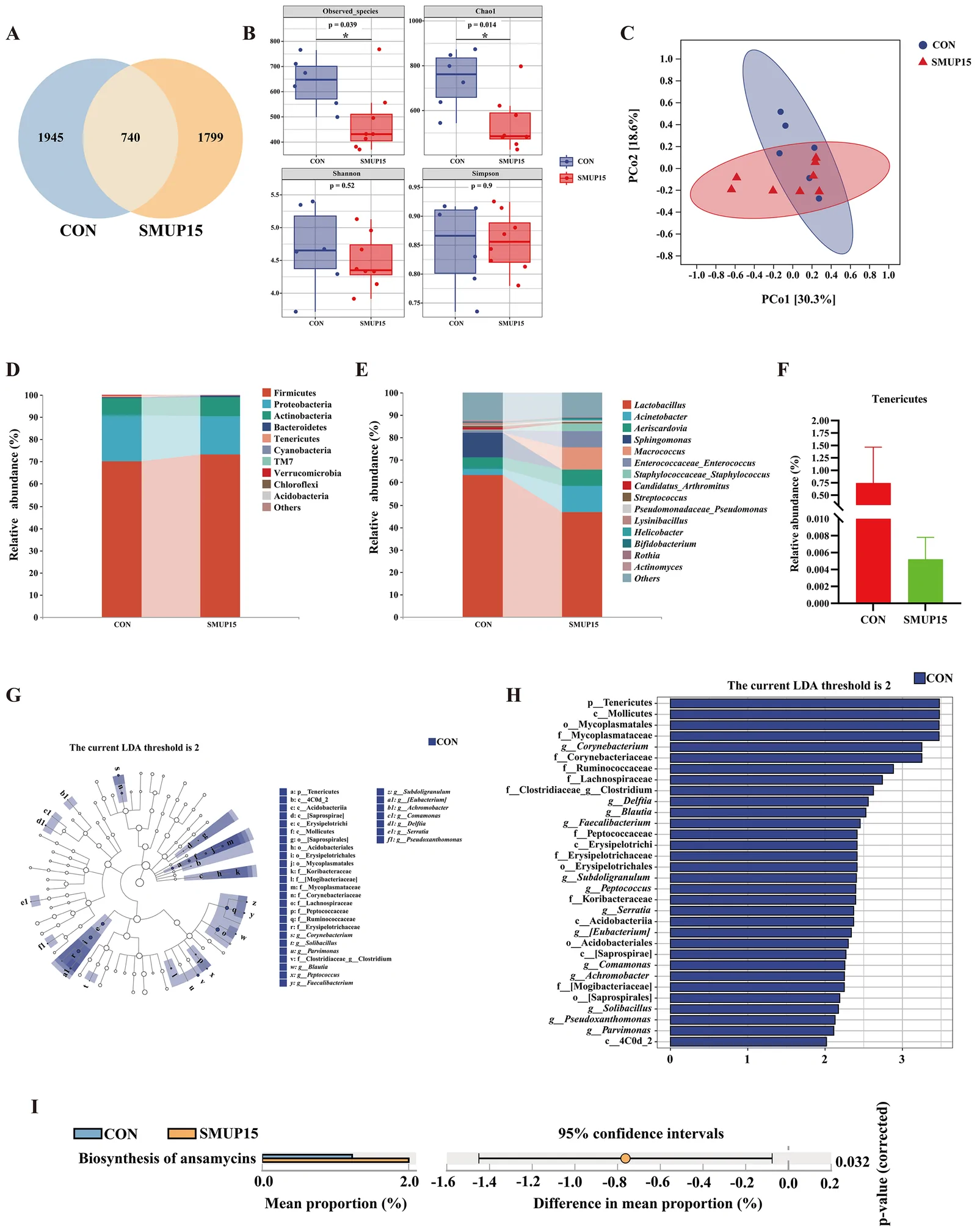
16S rDNA sequencing of ileal microbiota. **(A)** Venn diagram of ASVs. **(B)** Alpha diversity indexes. **(C)** Principal coordinates analysis (PCoA) based on Bray-Curtis distance. **(D)** Bar plot of the top 10 most abundant taxa at the phylum level. **(E)** Bar plot of the top 15 most abundant taxa at the genus level. **(F)** Bar plot of Tenericutes between the two groups. **(G)** Taxonomic cladogram of linear discriminant analysis combined with effect size measurements (LEfSe) analysis. **(H)** Histogram of LDA scores of the LEfSe analysis. **(I)** Comparison of predicted metabolic pathway abundance between the two groups by STAMP. ^*^P < 0.05. CON group, fed basal diet; SMUP15 group, fed basal diet supplemented with 15 g/kg *Salvia miltiorrhiza* ultrafine powder (SMUP).

A total of 4484 ASVs were detected, of which 2685 and 2539 ASVs were in the CON group and SMUP15 group, respectively. Among them, 740 ASVs were shared between these two groups, while 1945 ASVs were unique to the CON group, and 1799 ASVs were unique to the SMUP15 group (**Figure 3A**). The alpha-diversity indices of the ileal microbiota are presented in **Figure 3B**. Compared with the CON group, the SMUP15 group had decreased Observed_species (*P* < 0.05) and Chao1 (*P* < 0.05), indicating a reduction in microbial richness. The PCoA plot based on Bray-Curtis distance (**Figure 3C**) showed that PCo1 and PCo2 explained 30.3% and 18.6% of the total variation in the ileal microbiota, respectively. Samples from the two groups formed distinct clusters with partial overlap, indicating that dietary SMUP supplementation significantly altered the overall structure of the ileal microbial community in laying hens during the late laying period.

The composition and distribution of the ileal microbiota at the phylum level are shown in **Figure 3D**. Firmicutes, Proteobacteria, and Actinobacteria were the dominant phyla in both groups, collectively accounting for more than 98% of the total phyla. Compared with the CON group, the relative abundance of Firmicutes had an increasing trend in the SMUP15 group (69.99% vs. 73.12%), while that of Proteobacteria exhibited a decreasing trend (20.77% vs. 17.19%).

The composition of the ileal microbiota at the genus level is presented in **Figure 3E**. *Lactobacillus* was the dominant genus in both groups, accounting for 63.14% of the total genera in the CON group and 46.84% in the SMUP15 group. Compared with the CON group, the SMUP15 group showed an increase in the relative abundances of *Acinetobacter* (2.98% vs. 11.50%) and *Macrococcus* (0.02% vs. 10.14%). Trends of increased relative abundance were also observed for *Enterococcus*, *Staphylococcus*, and *Helicobacter* in the SMUP15 group. Meanwhile, the relative abundance of *Sphingomonas* was notably lower in the SMUP15 group (10.75% vs. 0.04%). The relative abundances of other genera, such as *Candidatus_Arthromitus* and *Lysinibacillus*, were generally lower, with *Lysinibacillus* being undetectable in the SMUP15 group. The taxonomic difference showed that the relative abundance of Tenericutes was lower (*P* < 0.05) in the SMUP15 group compared with the CON group (**Figure 3F**).

The linear discriminant analysis effect size (LEfSe) analysis was performed to identify bacterial taxa with significant differential abundance between the CON and SMUP15 groups, with an LDA threshold of 2. The taxonomic cladogram (**Figure 3G**) and corresponding histogram of LDA scores (**Figure 3H**) revealed that all identified biomarkers were enriched in the CON group. Specifically, taxa with LDA scores > 3, including the phylum Tenericutes, class Mollicutes, order Mycoplasmatales, family Mycoplasmataceae, genus Corynebacterium, and family Corynebacteriaceae, were significantly more abundant in the CON group. No bacterial taxa were identified as biomarkers enriched in the SMUP15 group. As shown in **Figure 3I**, PICRUSt2 functional prediction revealed that the biosynthesis of ansamycins pathway was significantly more abundant in the SMUP15 group than in the CON group (*P* < 0.05).

### 3.5 Pearson correlation and mantel test analysis

**Figure 4:**
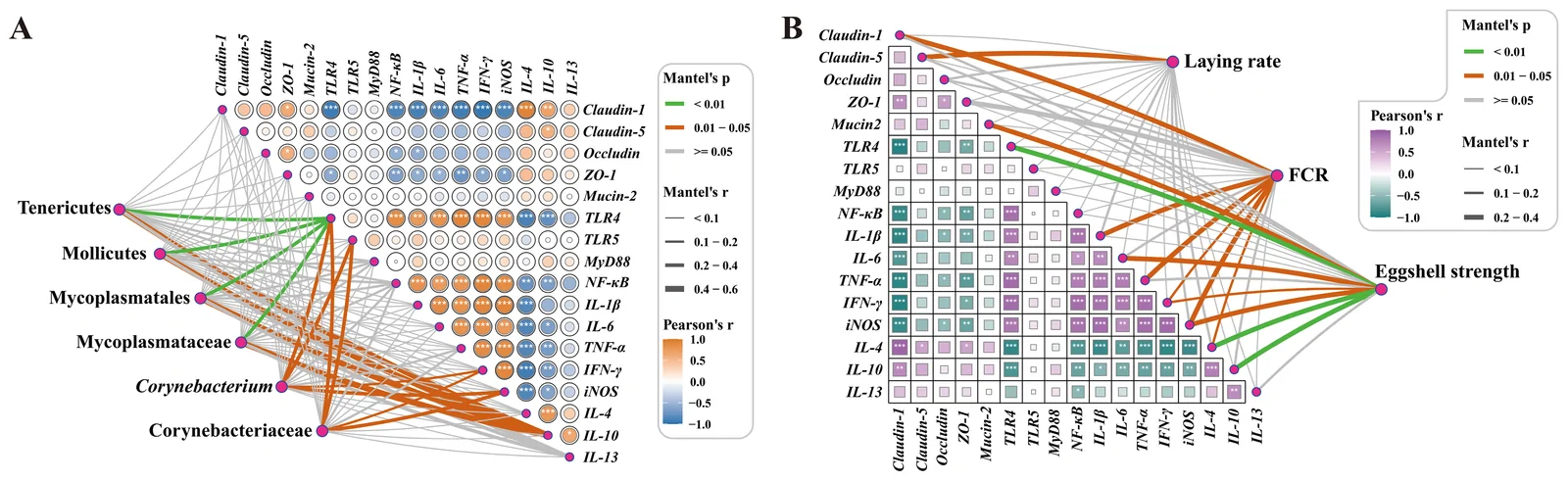
Pearson correlation and Mantel test analysis. **(A)** The upper right corner shows the Pearson correlation heatmap of ileal inflammation-related gene expression level, while the lower left corner displays the Mantel test network diagram between the biomarker microbe (LDA > 3) abundance matrix and the ileal inflammation-related gene expression level matrix. **(B)** The lower left corner shows the Pearson correlation heatmap of ileal inflammation-related gene expression level, while the upper right corner presents the Mantel test network diagram between laying performance/egg quality traits and the ileal inflammation-related gene expression level. In the Mantel test networks, the thickness of the lines represents different Mantel’s r values, and the color of the lines indicates different Mantel’s p values. ^*^P < 0.05; ^**^P < 0.01; ^***^P < 0.001.

The combined Pearson correlation and Mantel test analysis uncovered associations between biomarker taxa (LDA > 3) and the expression of ileal inflammation- and barrier-related genes (**Figure 4A**). Tight junction genes, including *claudin-1*, *occludin* and *ZO-1*, were negatively correlated with *TLR4*, *NF-κB*, *IL-1β*, *IL-6*, *TNF-α*, *IFN-γ* and *iNOS*. Taxa belonging to Tenericutes, Mollicutes, Mycoplasmatales, and Mycoplasmataceae were associated with the above inflammatory genes (*P* < 0.01; Mantel’s r = 0.2–0.6). Similarly, the *Corynebacterium* and Corynebacteriaceae showed significant correlations with pro-inflammatory cytokines (*IL-1β*, *IL-6*, *TNF-α*, *IFN-γ*, and *iNOS*) based on Mantel analysis. In addition, Tenericutes and its subordinate taxa (Mollicutes, Mycoplasmatales, and Mycoplasmataceae) had remarkable associations with *TLR4*, *MyD88*, *NF-κB*, and their downstream pro-inflammatory cytokines (*P* < 0.05), and subsequent Pearson analysis verified the positive direction of these correlations.

To further explore the functional implications of gut health-associated molecular signatures, the combined Pearson correlation and Mantel test were expanded to evaluate linkages between ileal barrier/inflammation gene expression and core laying performance as well as egg quality traits (**Figure 4B**). Mantel results revealed that *claudin-1* expression was associated with laying rate (*P* < 0.05), whereas *TLR4*, *IL-1β*, *IL-6*, *TNF-α*, *IFN-γ* and *iNOS* were correlated with FCR and eggshell strength. Of these genes, TLR4 displayed an association with eggshell strength (*P* < 0.01).

### 3.6 Differentially expressed gene and WGCNA analysis of the GSE278197 dataset

**Figure 5:**
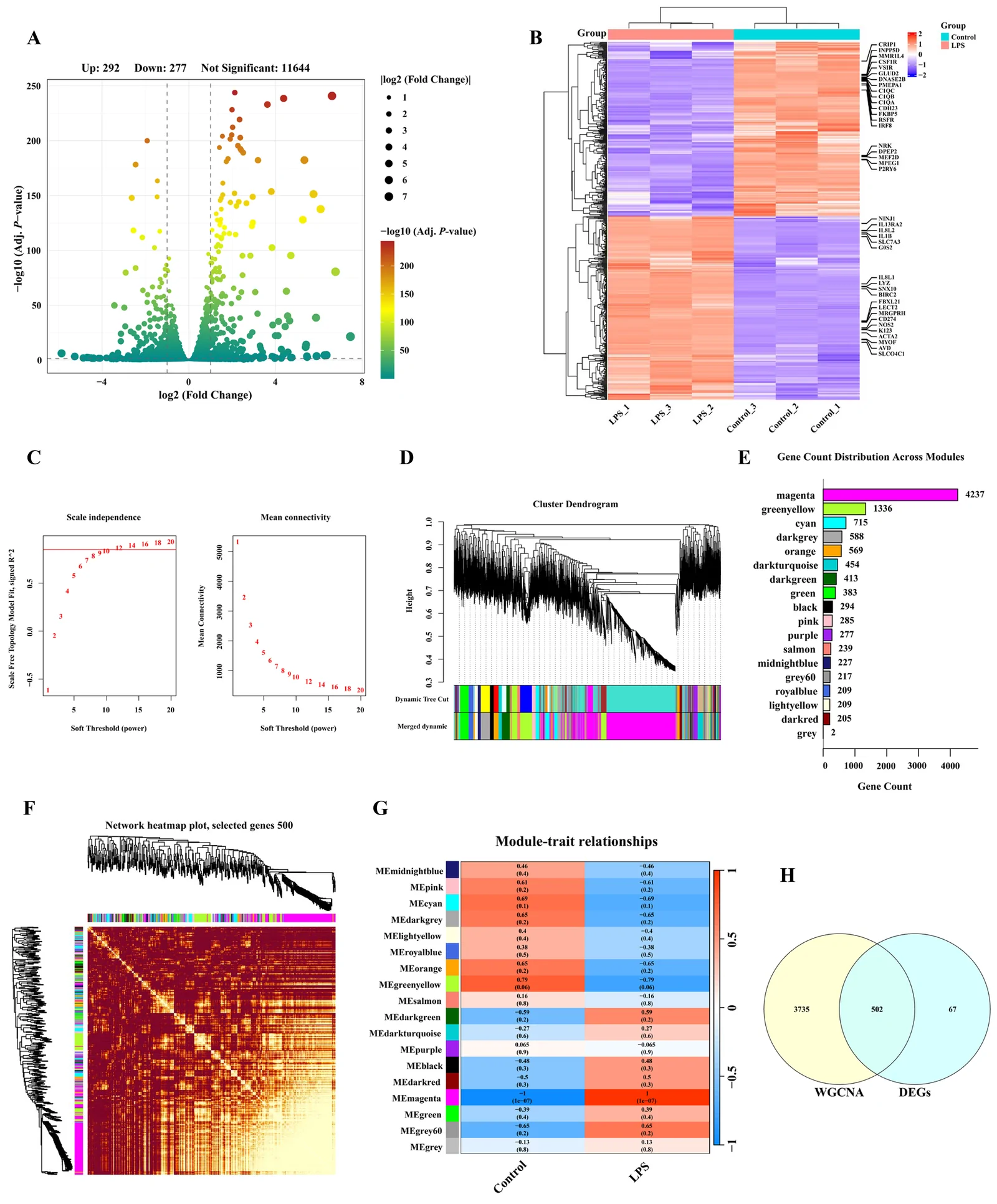
Screening of chicken macrophage polarization-related targets based on the GSE278197 dataset. **(A, B)** Differential expression analysis. **(A)** Volcano plot of differential expression analysis. Each dot represents one gene. The dot color shows a gradient from red to green; a color closer to red indicates a smaller adjusted *P*-value. Dot size represents the fold change, with larger dots indicating greater fold changes. **(B)** Hierarchical clustering heatmap of differentially expressed genes (DEGs). The top 20 most significantly upregulated and downregulated genes (sorted by adjusted *P*-value) are labeled. **(C, G)** Weighted gene co-expression network analysis (WGCNA). **(C)** Soft threshold power (β) selection. The left panel shows the scale-free topology model fit index (R²) corresponding to different soft threshold powers, with the red horizontal line indicating R² = 0.85. The right panel shows the mean connectivity of networks constructed with different soft threshold powers. **(D)** Upper panel: gene clustering tree generated based on the topological overlap dissimilarity matrix (1-TOM). Lower panel: different colors represent distinct gene modules. **(E)** Number of genes contained in each gene module. **(F)** Gene co-expression heatmap based on topological overlap matrix (TOM) values. **(G)** Module-trait/group correlation heatmap. **(H)** Venn diagram of overlapping results from differential expression analysis and WGCNA, showing 502 shared genes.

As shown in **Figure 5A**, the volcano plot displays the distribution of DEGs between two groups. Using the thresholds of |log_2_FC| > 1 and adjusted *P* < 0.05, 292 significantly upregulated genes and 277 significantly downregulated genes were identified, while 11,644 genes showed no significant expression changes. The most statistically significant and highly dysregulated genes are located at the upper extremes of the plot. The hierarchical clustering heatmap (**Figure 5B**) shows the expression profiles of all DEGs between the LPS and control groups. The top 20 most significantly upregulated and downregulated genes (ranked by adjusted *P*-value) showed a clear separation between the two groups, with samples clustering perfectly by treatment condition. LPS-treated samples exhibited consistent upregulation of pro-inflammatory-related genes and downregulation of immune regulatory-related genes compared with the controls, confirming the robust transcriptional response induced by LPS stimulation.

WGCNA analysis results are presented in **Figures 5C–G**. The soft threshold power (β) was determined based on scale-free topology and mean connectivity analysis (**Figure 5C**). The left panel shows the scale-free topology model fit index (R²) across different soft threshold powers, with the red horizontal line indicating R² = 0.85. As the power increased, R² gradually exceeded 0.85 and stabilized at β = 12, meeting the criteria for a scale-free network. The right panel displays the corresponding mean connectivity, which decreased with higher power, indicating reduced noise and fewer spurious connections. Based on these findings, a soft threshold power of β = 12 was selected to construct the gene co-expression network, ensuring both scale-free topology and adequate connectivity. The gene dendrogram (**Figure 5D**) was generated by hierarchical clustering using the topological overlap dissimilarity matrix (1-TOM). Initial gene modules were identified by dynamic tree cutting, and similar modules were subsequently merged using a dissimilarity threshold of 0.25 (corresponding to a correlation coefficient of 0.75). The top color bar shows the initial module assignments by dynamic tree cutting, while the bottom bar displays the final merged modules. The clustering tree clearly separates genes into distinct modules, and the merged modules show reduced redundancy and enhanced biological interpretability. As shown in **Figure 5E**, the gene distribution across merged WGCNA modules. The top five modules by gene number were included magenta (4237), green-yellow (1336), cyan (715), dark-gray (588), and orange (569). The gene co-expression heatmap based on the TOM for the top 500 selected genes is shown in **Figure 5F**. The distinct, clearly separated diagonal blocks indicate strong co-expression relationships among genes in the same module, while the darker background reflects weak correlations between different modules, confirming the modular structure of the co-expression network. The module-trait relationships heatmap (**Figure 5G**) illustrates the correlation between each module eigengene and the two groups (Control vs. LPS). The magenta module exhibited a positive correlation with the LPS group (*r* = 1) and a negative correlation with the control group (*r* = −1). As shown in the Venn diagram (**Figure 5H**), 502 genes were overlapped, which were defined as the key hub genes linking LPS-induced polarization to transcriptional changes in chicken macrophages.

### 3.7 Network pharmacology and molecular docking analysis

**Figure 6:**
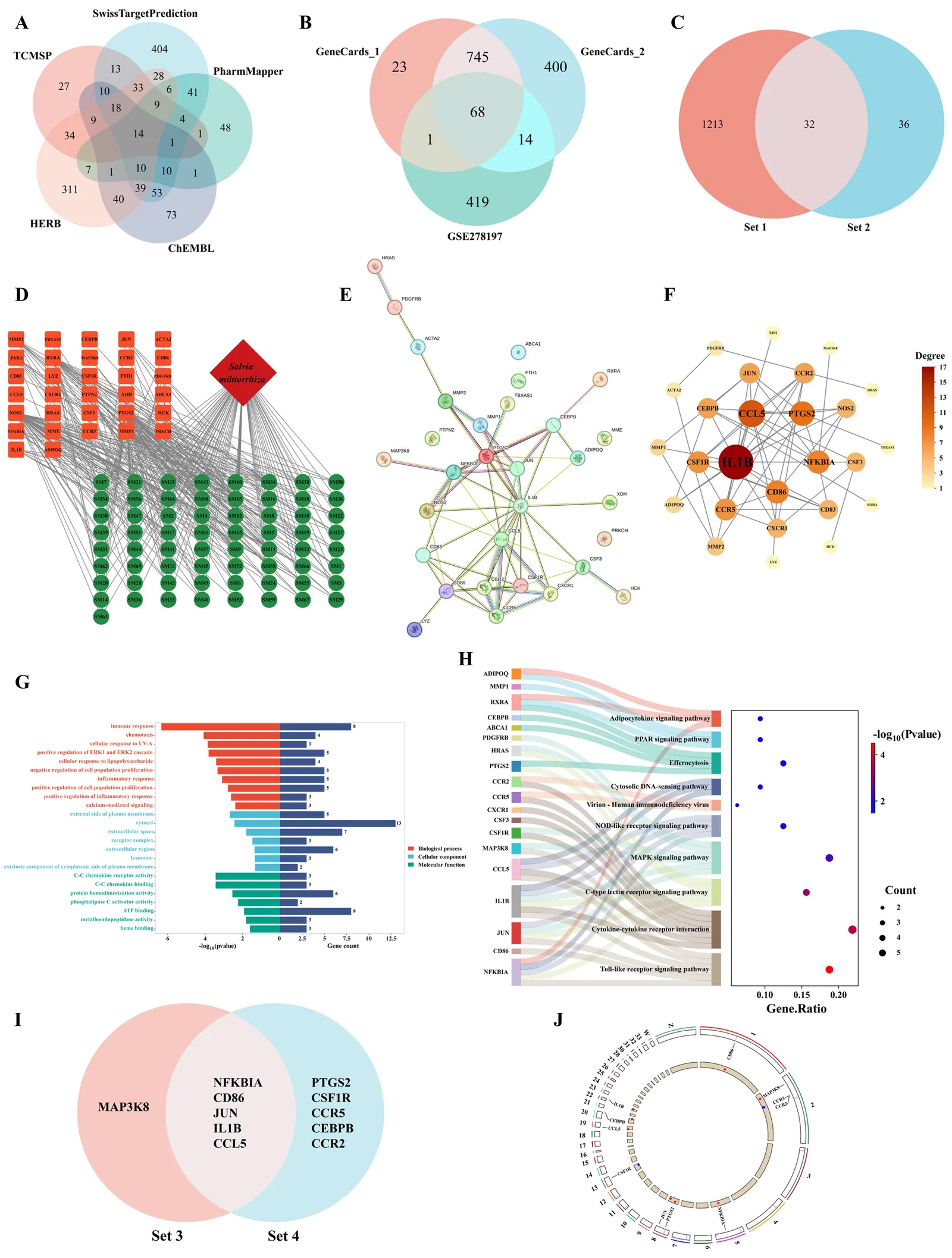
Network pharmacology analysis. **(A)** Venn diagram of potential bioactive compound targets of *Salvia miltiorrhiza* (SM) obtained from five different public databases. **(B)** Venn diagram for screening chicken macrophage polarization-related targets. GeneCards_1 represents targets retrieved from the GeneCards database using “*Gallus gallus macrophage polarization*” as the keyword, GeneCards_2 represents targets retrieved using “*Chicken macrophage polarization*” as the keyword, and GSE278197 represents chicken macrophage polarization-related targets screened based on the GSE278197 dataset. **(C)** Venn diagram showing the potential targets of SM components acting on chicken macrophage polarization. Set 1 represents the merged and deduplicated targets of potential bioactive compounds from SM obtained from five different public databases, and Set 2 represents chicken macrophage polarization-related targets. **(D)** Topological network diagram of SM–compound–target. Red diamonds represent SM, green circles represent potential bioactive compounds in SM, and orange rounded rectangles represent the intersection targets of Set 1 and Set 2. **(E)** A total of 32 intersection targets from Set 1 and Set 2 were imported into the STRING database to construct the protein–protein interaction (PPI) network. **(F)** The interaction relationships of the intersection targets of Set 1 and Set 2 obtained from the STRING database were visualized using Cytoscape 3.10.0 software. Each node represents one target. The node color shows a gradient from red to yellow; a color closer to red indicates a higher degree value. In addition, a larger node diameter and larger font size of the target label within the node also indicate a higher degree value. **(G)** Gene Ontology (GO) functional enrichment analysis of the 32 intersection targets of Set 1 and Set 2. **(H)** The Sankey diagram of Kyoto Encyclopedia of Genes and Genomes (KEGG) pathway enrichment analysis of the 32 intersection targets. **(I)** Set 3 represents the targets enriched in the toll-like receptor signaling pathway among the 32 intersection targets of Set 1 and Set 2, Set 4 represents the top 10 targets with the highest degree values in the PPI analysis. **(J)** Genomic locations of all target genes in Set 3 and Set 4 and their expression levels in the GSE278197 dataset. Red dots indicate significantly upregulated genes, and blue dots indicate significantly downregulated genes (LPS group vs CON group).

A total of 69 potential bioactive compounds of SM were identified in this study (**Supplementary Table 3**). Among these, 62 compounds were retrieved from the TCMSP database, and an additional 7 compounds were obtained based on the *Pharmacopoeia of the People’s Republic of China* (8) and published literature (22–25). To identify the targets of these 69 potential bioactive compounds, target prediction was performed using five public databases separately. As shown in **Figure 6A**, 173, 693, 279, 155, and 599 targets were retrieved from the TCMSP, SwissTargetPrediction, ChEMBL, PharmMapper, and HERB databases, respectively. After merging and removing redundant entries, a total of 1247 non-redundant targets were identified. Then, the GeneCards database was searched using the keywords “*Gallus gallus macrophage polarization*” and “*Chicken macrophage polarization*”, retrieving 837 and 1227 targets, respectively. These targets were intersected with the 502 hub genes identified from the GSE278197 dataset, yielding 68 overlapping targets (**Figure 6B**). These 68 targets were defined as the FTMPs of chicken. Subsequently, the FTMPs were intersected with the 1247 targets of the bioactive compounds of SM, resulting in 32 overlapping targets (**Figure 6C** and **Supplementary Table 4**). These 32 targets were defined as the PTSMs.

Next, a SM–compound–target network was constructed based on the PTSMs (**Figure 6D**). Degree centrality analysis of the network was performed, and the top 15 compounds with the highest degree values were selected as potential key compounds. The degree, molecule name and formula, and structural formula of these compounds are listed in **Table 2**. Afterward, the PTSMs were uploaded to the STRING database to construct a PPI network (**Figures 6E, F**). The top 10 targets, including IL1B, CCL5, PTGS2, CD86, NFKBIA, CSF1R, CCR5, CEBPB, JUN, and CCR2, with the highest degree values in the PPI network were identified as potential key targets (**Table 3**).

**Table 2:** Top 15 potential key compounds (ranked by degree value) in the *Salvia miltiorrhiza*–compound–macrophage polarization-related target network.

No.	Molecule name	Degree	Molecular formula	Structural formula
SM15	Baicalin	11	C_21_H_18_O_11_	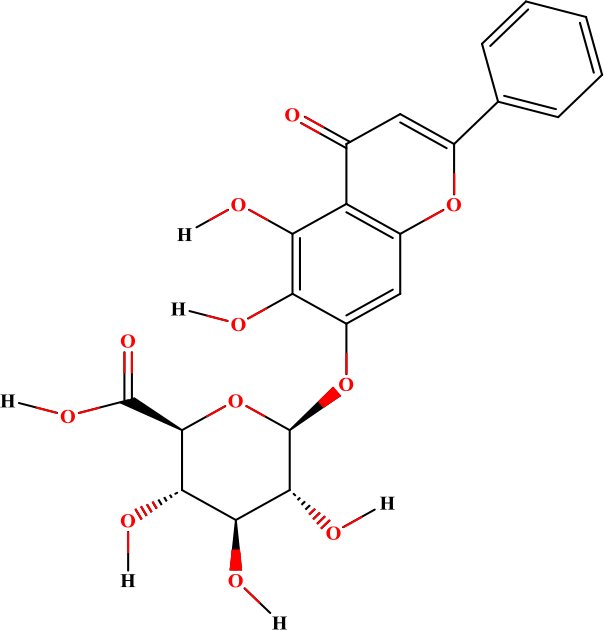
SM34	Luteolin	11	C_15_H_10_O_6_	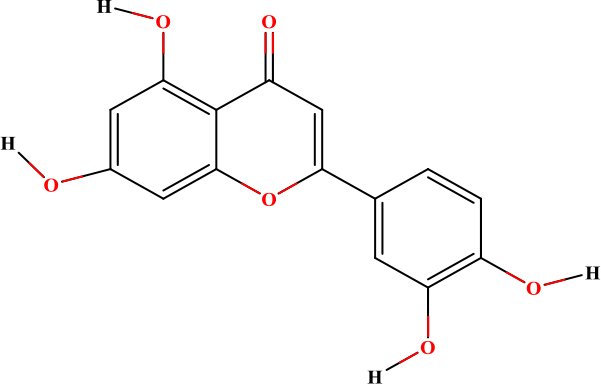
SM11	3-β-Hydroxymethyllenetanshiquinone	10	C_18_H_14_O_4_	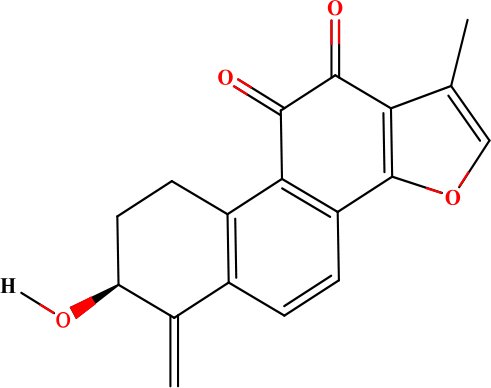
SM9	2-(4-hydroxy-3-methoxyphenyl)-5-(3-hydroxypropyl)-7-methoxy-3-benzofurancarboxaldehyde	9	C_20_H_20_O_6_	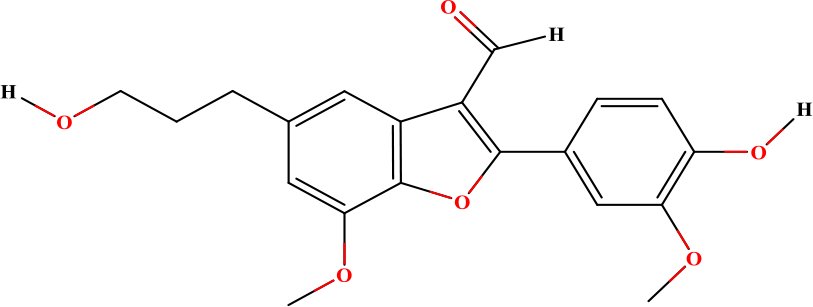
SM17	Cryptotanshinone	9	C_19_H_20_O_3_	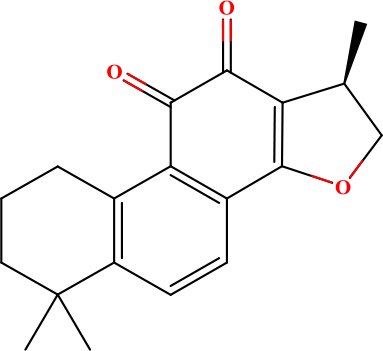
SM54	Rosmarinic acid	9	C_18_H_16_O_8_	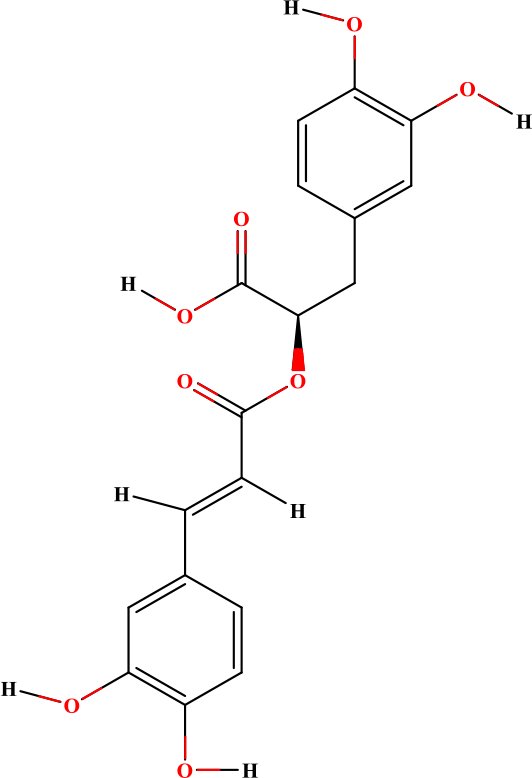
SM68	Tanshinone II A	7	C_19_H_18_O_3_	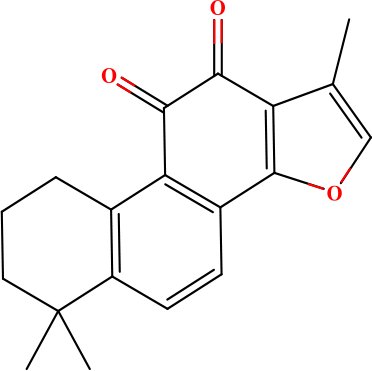
SM47	Protocatechualdehyde	7	C_7_H_6_O_3_	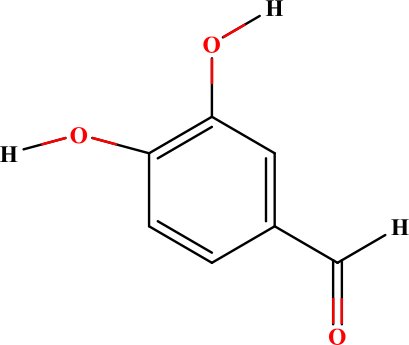
SM2	Tanshinone II B	6	C_19_H_18_O_4_	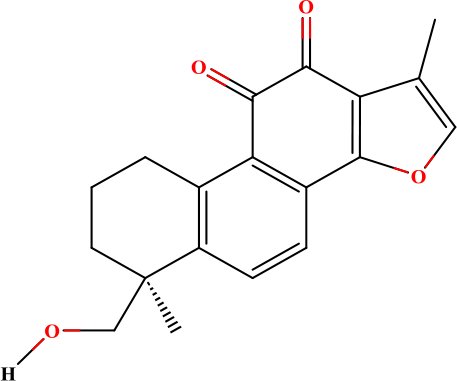
SM3	Tanshindiol A	6	C_18_H_16_O_5_	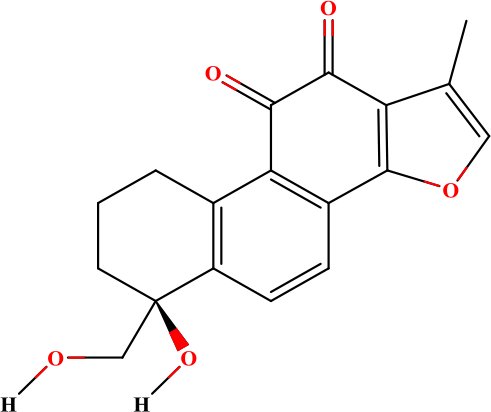
SM43	Neocryptotanshinone II	6	C_17_H_18_O_3_	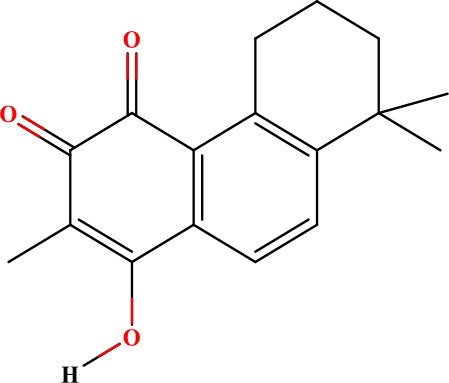
SM49	Przewalskin B	6	C_20_H_26_O_4_	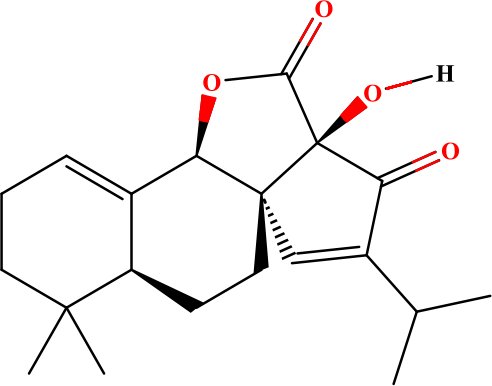
SM55	Salvianolic acid A	6	C_26_H_22_O_10_	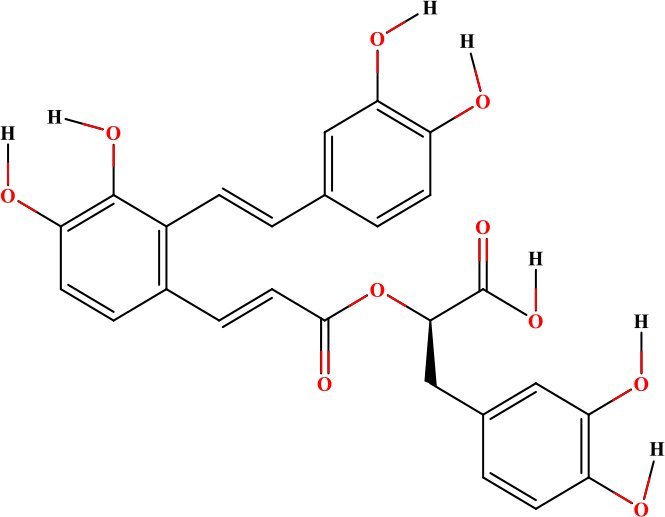
SM56	Salvianolic acid B	6	C_36_H_30_O_16_	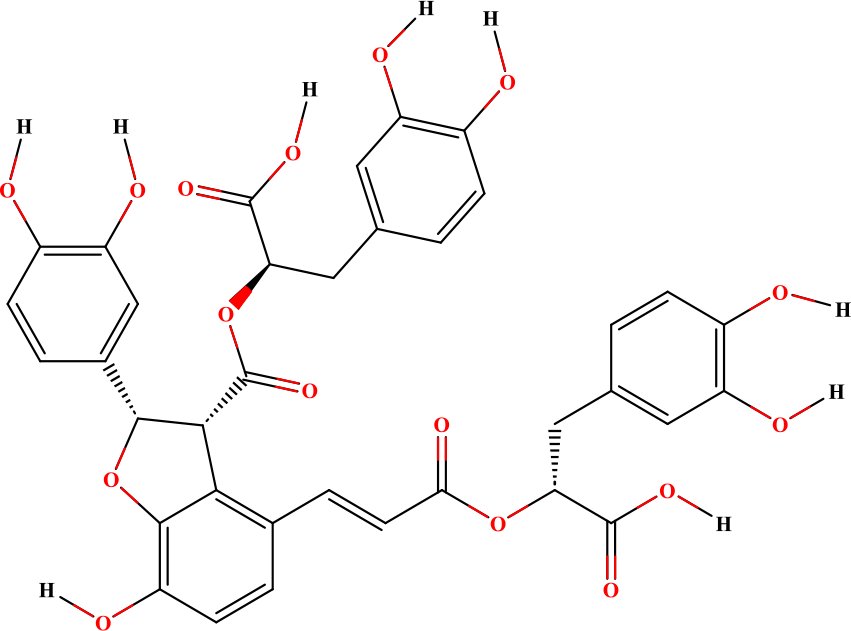
SM60	Salvilenone I	6	C_18_H_22_O_2_	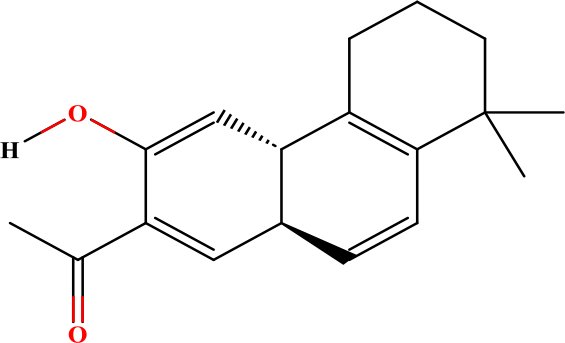

**Table 3:** Top 10 potential key targets (ranked by degree value) in protein–protein interaction (PPI) network analysis.

Target name	Full name	Degree	Betweenness	Closeness
IL1B	Interleukin-1β	17	291.80	0.03
CCL5	C-C motif chemokine 5	11	46.90	0.02
PTGS2	Prostaglandin-endoperoxide synthase 2	9	85.50	0.02
CD86	T-lymphocyte activation antigen CD86	8	51.57	0.02
NFKBIA	NF-κB inhibitor α	8	56.90	0.02
CSF1R	Macrophage colony-stimulating factor 1 receptor	7	7.57	0.02
CCR5	C-C chemokine receptor type 5	7	3.30	0.02
CEBPB	CCAAT/enhancer-binding protein β	6	50.00	0.02
JUN	Transcription factor Jun	6	7.33	0.02
CCR2	C-C motif chemokine receptor 2	6	1.40	0.02

Subsequently, the PTSMs were imported into the DAVID database for GO and KEGG enrichment analysis. The GO enrichment analysis of PTSMs (**Figure 6G**) revealed that the most enriched terms in BP were immune response, chemotaxis, cellular response to LPS, and inflammatory response, with the immune response term showing the highest gene count (*n* = 8). In the CC category, terms such as cytosol, extracellular space, external side of plasma membrane, and extracellular region were predominantly enriched, with cytosol exhibiting the highest gene count (*n* = 13). In the MF category, key enriched terms included ATP binding, protein homodimerization activity, C-C chemokine receptor activity, and C-C chemokine binding, with ATP binding showing the highest gene count (*n* = 8).

The KEGG pathway enrichment analysis of the 32 PTSMs (**Figure 6H**; **Supplementary Table 5**) identified the top 10 pathways, ranked by *P*-value. The TLR signaling pathway emerged as the most significantly enriched pathway (*P* = 3.15 × 10^-5^), with a gene ratio of 18.75%, involving six key targets (NFKBIA, CD86, JUN, IL1B, CCL5, and MAP3K8). This pathway was highlighted as the core regulatory axis in the Sankey diagram, showing direct connections to pivotal genes involved in innate immune recognition and inflammatory response. Other enriched pathways included cytokine-cytokine receptor interaction (*P* = 1.98 × 10^-4^, gene ratio = 21.88%), C-type lectin receptor signaling pathway (*P* = 5.32 × 10^-4^), and MAPK signaling pathway (*P* = 4.77 × 10^-3^), which are closely linked to macrophage polarization and inflammatory regulation.

To prioritize the most functionally relevant targets within the TLR signaling pathway, the six PTSMs enriched in TLR signaling were intersected with the top 10 targets identified via degree centrality analysis of the PTSMs PPI network (**Figure 6I**). The resulting five overlapping genes—NFKBIA, CD86, JUN, IL1B, and CCL5—were defined as CCTs.

**Figure 7:**
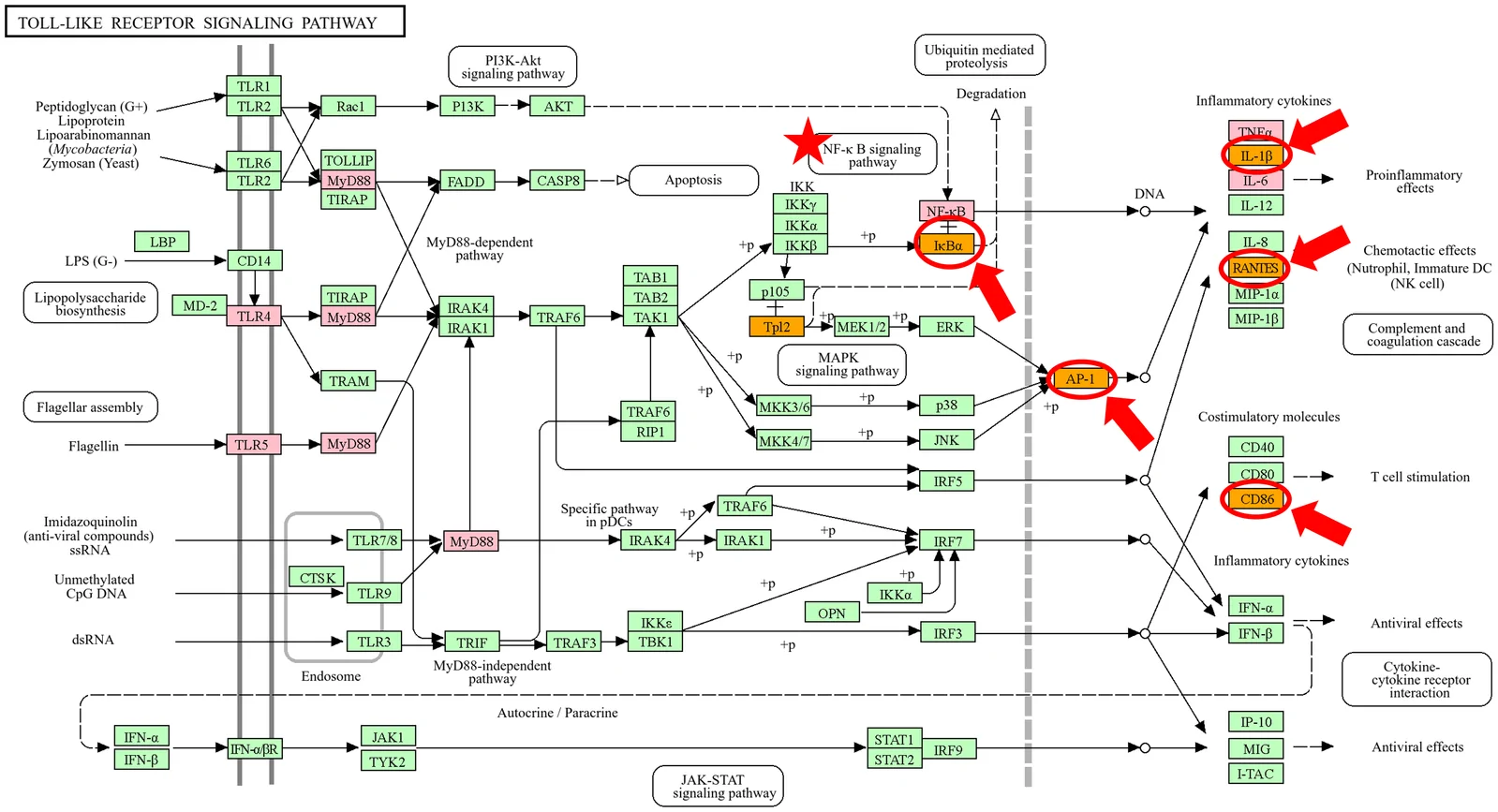
Detailed network of “toll-like receptor signaling pathway”. Genes highlighted in orange are the six genes enriched in the toll-like receptor signaling pathway among the 32 intersection target genes of Set 1 and Set 2, including Tpl2 (MAP3K8), IκBα (NFKBIA), AP-1 (JUN), IL-1β (IL1B), RANTES (CCL5), and CD86. Among them, the five intersection target genes from **Figure 6I** are labeled with red circles and arrows. Genes highlighted in pink are other genes involved in the RT-qPCR in this study. A sub-pathway of the toll-like receptor signaling pathway, NF-κB signaling pathway, is marked with a red five-pointed star.

**Figure 7** presents the detailed molecular network of the TLR signaling pathway, illustrating the localization of the six pathway-enriched PTSMs: Tpl2 (MAP3K8), IκBα (NFKBIA), AP-1 (JUN), IL-1β (IL1B), RANTES (CCL5), and CD86. These genes are highlighted in orange, with the five CCTs from **Figure 6I** further emphasized by red circles and arrows.

To further clarify the interactions between key bioactive compounds of SM and the core target NFKBIA, as well as to verify their regulatory effects, molecular docking experiments were performed in this study. Given that no experimentally determined crystal structure of chicken NFKBIA is available in the PDB database, two structural models were employed as receptors for cross-validated molecular docking to improve result reliability and ensure analytical rigor: one was the experimentally resolved 3D crystal structure of human NFKBIA (PDB ID: 1NFI) downloaded from the PDB database, and the other was the homology-based 3D structure of chicken NFKBIA predicted by AlphaFold 3 based on the sequence obtained from the UniProt database (UniProt ID: Q91974). This cross-species dual-receptor design not only ensures the baseline reliability of docking analysis using the experimentally validated human structure, but also recapitulates the native protein conformational features of the target species, providing a rigorous cross-validation basis for the subsequent in-depth analysis of compound–target interactions. The compounds (ligands) used for molecular docking were the 15 potential key compounds mentioned above.

**Figure 8:**
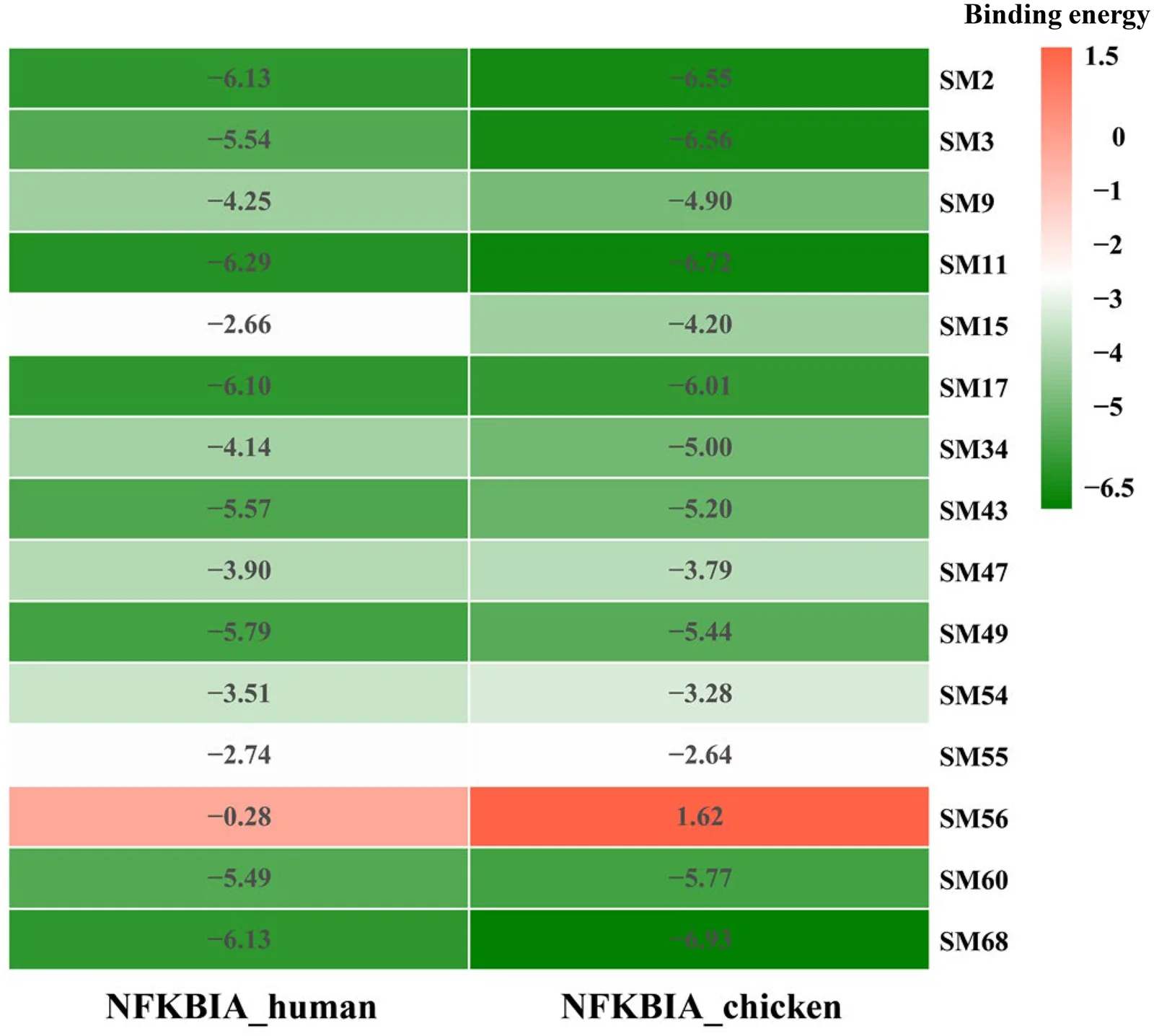
Heatmap of molecular docking binding energies. NFKBIA_human: Experimentally determined structure of human NFKBIA protein downloaded from the PDB database (PDB ID: 1NFI); NFKBIA_chicken: Three-dimensional structure of chicken NFKBIA protein predicted by the artificial intelligence tool AlphaFold 3, based on the coding sequence downloaded from the UniProt database (UniProt ID: Q91974). SM2, tanshinone II B; SM3, tanshindiol A; SM9, 2-(4-hydroxy-3-methoxyphenyl)-5-(3-hydroxypropyl)-7-methoxy-3-benzofurancarboxaldehyde; SM11, 3-β-hydroxymethyllenetanshiquinone; SM15, baicalin; SM17, cryptotanshinone; SM34, luteolin; SM43, neocryptotanshinone II; SM47, protocatechualdehyde; SM49, przewalskin B; SM54, rosmarinic acid; SM55, salvianolic acid A; SM56, salvianolic acid B; SM60, salvilenone I; SM68, tanshinone II A.

**Figure 9:**
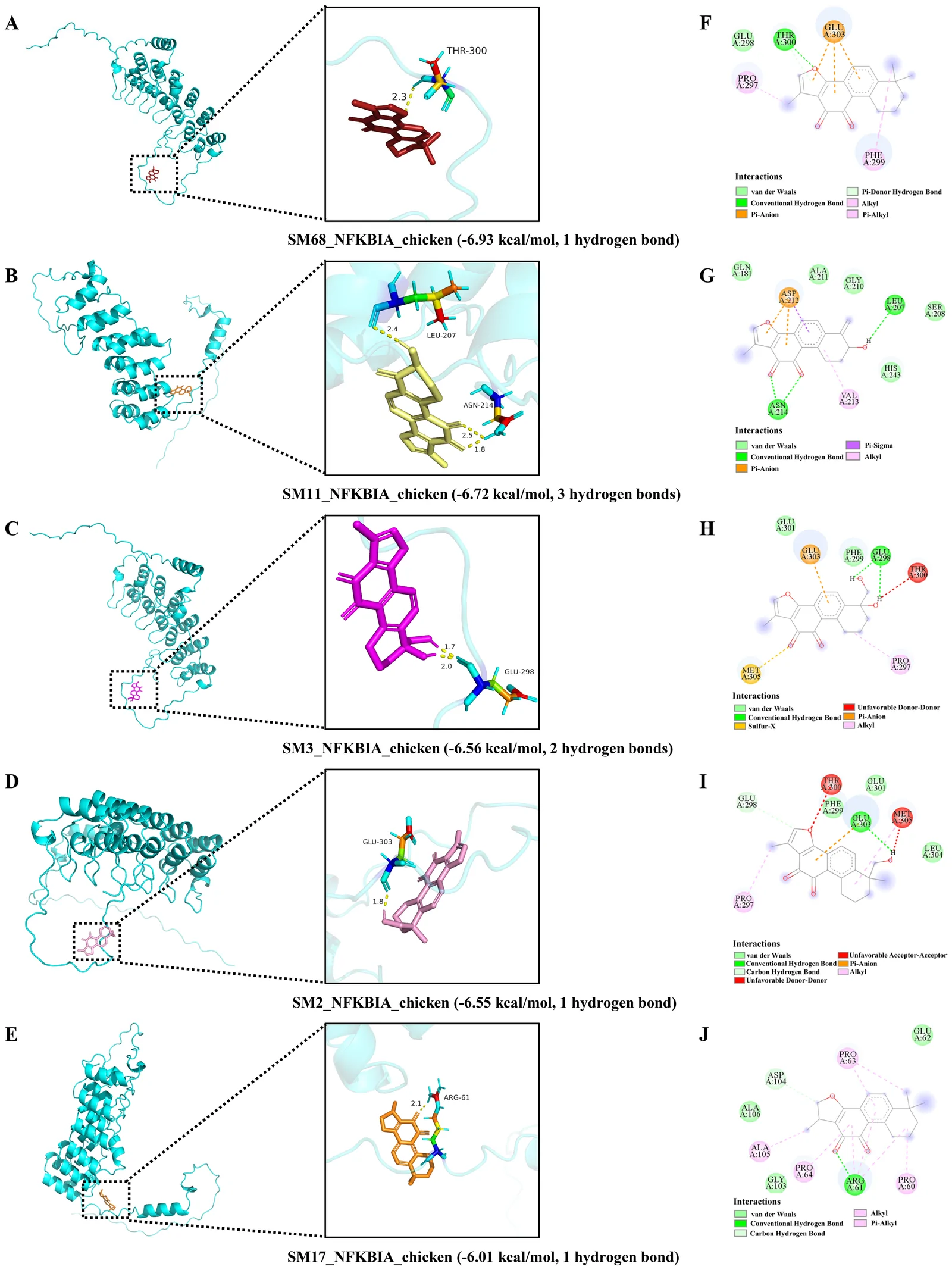
3D and 2D schematic diagrams of molecular docking results. **(A–E)** 3D structures of the predicted chicken NFKBIA protein docked with SM68 (tanshinone II A), SM11 (3-β-hydroxymethyllenetanshiquinone), SM3 (tanshindiol A), SM2 (tanshinone II B), and SM17 (cryptotanshinone), respectively. The left panels show the binding positions of the compound ligands with the protein receptor, while the right panels provide magnified views of the binding sites to illustrate the detailed interactions between the ligands and the receptor. **(F–J)** 2D structures of NFKBIA protein docked with the corresponding ligands, clearly depicting various interactions between the receptor and ligands, such as hydrogen bonds and van der Waals forces.

As shown in the binding energy heatmap (**Figure 8**), the binding energy profiles of the 15 compounds were highly consistent between the human and chicken NFKBIA models, with differences in binding energies of < 1 kcal/mol for most compounds. Among all tested compounds, SM68 (tanshinone II A) exhibited the strongest binding potential, with binding energies of −6.93 kcal/mol for chicken NFKBIA and −6.13 kcal/mol for human NFKBIA, indicating highly stable interactions between tanshinone II A and NFKBIA. To further illustrate the binding patterns, the top five pairs of chicken NFKBIA–compound complexes with the lowest binding energies were visualized in 3D and 2D (**Figure 9**). Among these, tanshinone II A (**Figures 9A, F**) formed a direct interaction with key amino acid residues in the receptor active pocket via one hydrogen bond, accompanied by extensive van der Waals forces that filled the hydrophobic cavity, resulting in a low-energy and stable binding conformation.

### 3.8 Molecular dynamics simulations

**Figure 10:**
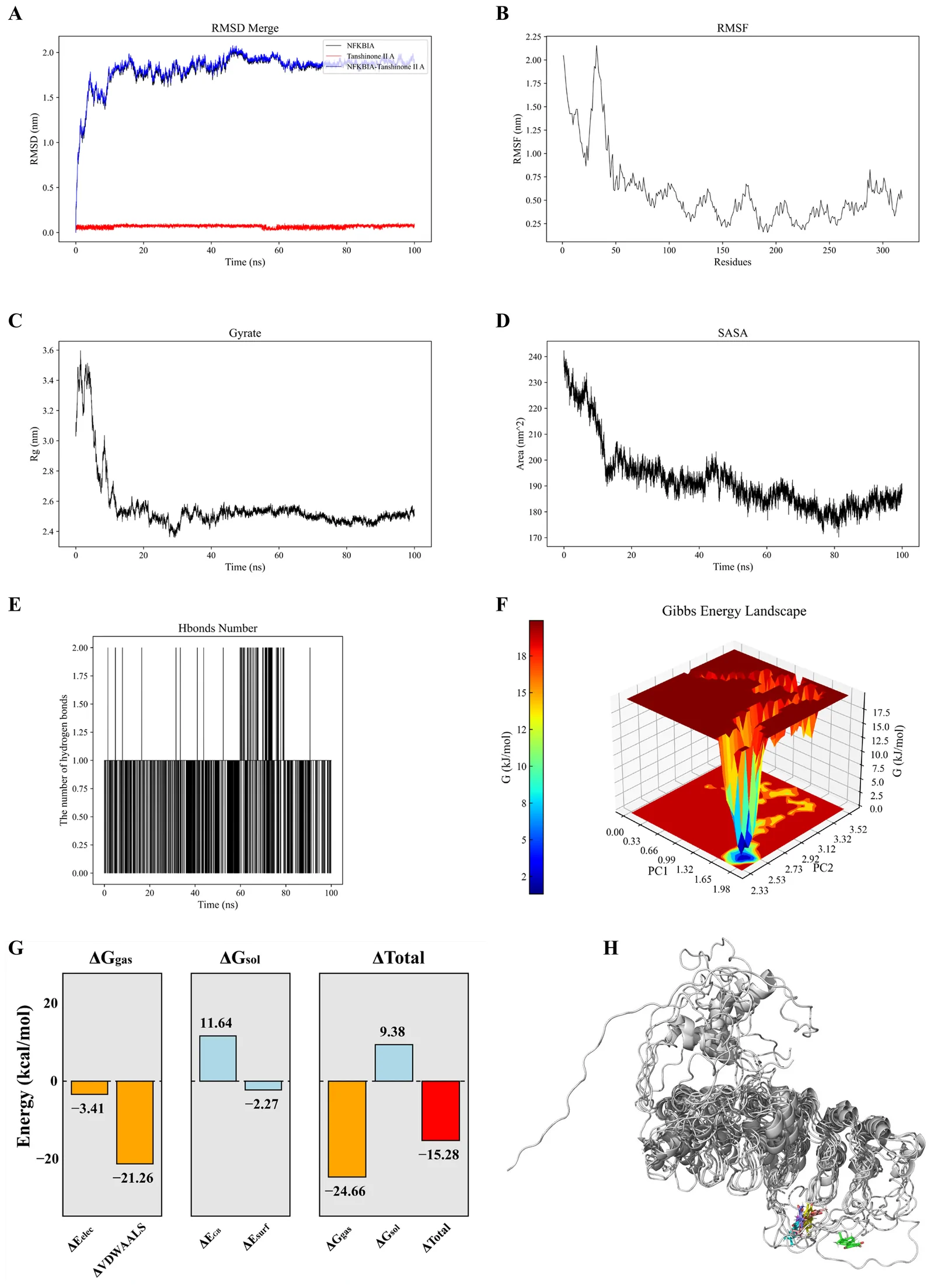
100 ns molecular dynamics simulation of the molecular docking complex of tanshinone IIA and the AI-predicted structure of chicken NFKBIA protein. **(A)** Root mean square deviation (RMSD) curves for heavy (non-hydrogen) atoms of tanshinone II A, Cα backbone of the NFKBIA protein, and the Cα backbone of the protein within the complex. **(B)** Root mean square fluctuation (RMSF) curve for the complex protein Cα backbone. **(C)** Radius of gyration (Rg) curve for the complex protein Cα backbone. **(D)** Solvent accessible surface area (SASA) curve for the complex protein Cα backbone. **(E)** Variation in hydrogen bond number of the NFKBIA-tanshinone II A complex during molecular dynamics simulation. **(F)** Free energy landscape of the NFKBIA-tanshinone II A complex during molecular dynamics simulation. **(G)** Average binding free energy of the NFKBIA-tanshinone II A complex during molecular dynamics simulation, *ΔVDWAALS*, *ΔE_ele_*, *ΔE_GB_*, *ΔE_surf_*, *ΔG_gas_*, *ΔG_sol_*, and *ΔTotal* represent van der Waals energy, electrostatic energy, polar solvation energy, nonpolar solvation energy, gas-phase free energy, solvation free energy, and average binding free energy, respectively. **(H)** Comparison of binding conditions for the NFKBIA-tanshinone II A complex at six molecular dynamics simulation time points (0, 20, 40, 60, 80, and 100 ns). The NFKBIA protein is shown in gray, and the small molecules in green, cyan, purple, yellow, light-rose, and purple-blue represent tanshinone IIA at 0, 20, 40, 60, 80, and 100 ns, respectively.

To further validate the molecular docking results, MD simulation was performed on the receptor–ligand complex with the lowest binding energy, specifically chicken NFKBIA and tanshinone II A (**Figure 10**). As shown in **Figure 10A**, the RMSD of NFKBIA protein Cα backbone reached equilibrium at ~20 ns and stabilized at 1.861 ± 0.068 Å throughout the MD simulation. Meanwhile, the RMSD of ligand tanshinone II A remained consistently very low level (< 0.5 Å), indicating no significant displacement or conformational change within the binding pocket. The RMSD of the NFKBIA–tanshinone II A complex also reached equilibrium after 20 ns, converging at 1.879 ± 0.064 Å. These results collectively demonstrate that tanshinone II A forms a stable binding interaction with NFKBIA, without flipping or large-scale movement, thereby strongly validating the reliability of the molecular docking prediction.

The RMSF quantifies the positional flexibility of individual residues during MD simulations, where lower values indicate reduced structural fluctuation and higher stability. The RMSF curve of NFKBIA Cα backbone in the NFKBIA-tanshinone II A complex is shown in **Figure 10B**. The RMSF values of most residues, including those in the ligand-binding pocket, were consistently lower, with the majority falling below 0.7 nm and many in the range of 0.15–0.5 nm. The lowest RMSF value was observed at residue 192 (0.155 nm), and residues in the binding interface region mostly exhibited values below 0.5 nm, indicating that the binding pocket remained high rigidit throughout the MD simulation. Notably, the highest RMSF values occurred in the N-terminal region (residues 1–45), peaking at 2.154 nm for residue 32. Other flexible regions with moderate RMSF values included residues 285–305, ranging from 0.448 to 0.828 nm. However, these regions corresponding naturally flexible loops located far from the ligand-binding site and did not affect the stability of the NFKBIA-tanshinone II A complex. Overall, the RMSF results confirm that the NFKBIA maintained a stable conformation during the simulation and the binding interface with tanshinone II A remained rigid, further supporting the high stability of the complex.

The Rg​ is commonly used to characterize the compactness and stability. A larger Rg​ indicates a more expanded conformation, whereas a smaller and stable Rg​ reflects a tightly packed and stable structure. The Rg​ curve of the NFKBIA Cα backbone in the docked complex is shown in **Figure 10C**. During the initial phase (0–15 ns), the Rg​ decreased rapidly from approximately 3.6 nm, followed by a conformational relaxation and adjustment period between 15–35 ns. From 35 ns to the end of the 100 ns simulation, the Rg​ remained stable around 2.5 nm, fluctuating within ± 0.05 nm, and showing no significant continuous upward or downward trend.

The SASA curve of NFKBIA in the docked complex is shown in **Figure 10D**. During the initial simulation phase (0–15 ns), the SASA decreased rapidly from approximately 242 nm², and then remained within the range of 170–205 nm² throughout the subsequent 15–100 ns.

The variation in the number of hydrogen bonds in the NFKBIA–tanshinone II A complex during the 100 ns simulation is shown in **Figure 10E**. Throughout the simulation, the number of hydrogen bonds remained stable between 0 and 2, with the majority of the simulation time maintaining 1 hydrogen bond, and only transiently forming 2 hydrogen bonds in several periods.

The Gibbs free energy landscape of the NFKBIA–tanshinone II A complex is shown in **Figure 10F**. The energy landscape exhibits a nearly single, well-defined low-energy basin without obvious multi-cluster distribution or energy barriers. This result indicates that the complex remained concentrated in a single thermodynamically favorable conformational state throughout the 100 ns simulation, with highly compact conformational distribution and no frequent conformational transitions or state dissociation. These findings are consistent with the overall stability reflected by the RMSD and Rg​ curves, further confirming the stable interaction between NFKBIA and tanshinone II A.

To further quantify the binding affinity between NFKBIA and tanshinone II A, the average binding free energy of the complex and its energetic components were calculated using the molecular mechanics/generalized Born surface area (MM/GBSA) method (**Figure 10G**). The results showed that the total binding free energy (*ΔTotal*) of the NFKBIA–tanshinone II A complex was –15.28 kcal/mol, indicating a high binding affinity of the complex, which is highly consistent with the molecular docking prediction. The conformations of the NFKBIA–tanshinone II A complex at six time points (0, 20, 40, 60, 80, and 100 ns) during the MD simulation were extracted and superimposed (**Figure 10H**). The animation of the molecular dynamics simulation process (100 frames) was shown in **Supplementary Video S1**.

## 4 Discussion

Decline in laying performance and deterioration in egg quality are common occurrences in hens during the late laying period. Our previous study found that dietary supplementation with 15 g/kg SMUP during late laying period improved laying rate, reduced FCR, and enhanced eggshell strength of hens (11). The present study further indicated that dietary SMUP improves intestinal health of late-laying hens by strengthening the intestinal barrier, alleviating inflammation, and modulating the gut microbiota. These improvements in intestinal health supported SMUP as a promising natural plant-derived feed additive for hens during late laying period.

The main functions of the intestinal epithelium included absorption of nutrients and protection against lumen pathogens (28). Notably, CD and VCR are sensitive indicators of intestinal inflammatory status. Increased CD and reduced VCR are associated with higher numbers of intraepithelial CD3^+^ T-lymphocytes, reflecting local inflammation and compromised gut health (29). The present study found that dietary SMUP supplementation improved the ileal morphology of laying hens, as evidenced by reduced CD and increased VCR. Consistent with our findings, dietary 1.5 g/kg SM polysaccharide (SMP) improved intestinal morphology and reduced local inflammation by increasing ileal VH and VCR but decreasing CD in weaned piglets, indicating improved intestinal morphology and reduced local inflammation (30). Additionally, salvianolic acid B administration decreased both ileal VH and CD, while having no effect on VCR in a rat ischemia-reperfusion model (31). These discrepancies may be attributed to differences in animal species, experimental models, and the form of SM used.

Tight junctions (TJs) are composed of transmembrane proteins (e.g., occludin and claudins) and intracellular scaffolding proteins (e.g., ZO family proteins). When TJ’s integrity is compromised, paracellular permeability increases, leading to translocation of luminal harmful substances into the internal environment, which activates the mucosal immune system and subsequently triggers sustained inflammatory responses and tissue damage (32). In the present study, dietary SMUP treatment upregulated the expressions of *claudin-1*, *occludin*, and *ZO-1*. A previous study showed that SM strengthened the intestinal barrier in diabetic mice by upregulating *ZO-1*, *occludin*, and *claudin-5* expressions in both the ileum and colon (33). Similarly, tanshinone IIA was shown to ameliorate the downregulating effect on *claudin-1*, *claudin-3*, *ZO-1*, and *occludin* expressions induced by deoxynivalenol in IPEC-J2 cells (34), and also upregulated *occludin*, *claudin-1*, and *ZO-1* expressions in the jejunal mucosa of a pig ischemic stroke model (35). Taken together, these results indicate that SMUP15 effectively preserves intestinal tight junction integrity and enhances the mechanical barrier function in hens during late laying period. These observed protective effects may be attributed to bioactive compounds in SM, such as tanshinone IIA.

Intestinal macrophages, which are indispensable in innate immunity, are highly plastic, polarizing into pro-inflammatory M1 or anti-inflammatory M2 phenotypes depending on microenvironmental cues (36, 37). Impaired intestinal epithelium or tight junctions enable luminal bacteria to penetrate into the lamina propria, and then LPS derived from the gram-negative bacteria is recognized by macrophage-surface TLR4 to trigger the activation of transcription factor NF-κB. Then, the activation of TLR4/NF-κB pathway drives M1 polarization and pro-inflammatory cytokine production (e.g., IL-1β, IL-6, and TNF-α), whereas IL-4, IL-10, and IL-13 promote M2 polarization and resolution of inflammation (38–40). In the present study, dietary SMUP downregulated the expressions of *TLR4*, *NF-κB p65*, and pro-inflammatory cytokines (*IL-1β*, *IL-6*, *TNF-α*, *IFN-γ*, and *iNOS*), while upregulating the expressions of anti-inflammatory *IL-4* and *IL-10* in ileal tissue, alongside increased plasma IL-10 level. Numerous studies involving SM extracts are consistent with our findings. For instance, total tanshinones exert anti-inflammatory effects by blocking TLR4 dimerization and inhibiting the TLR4/NF-κB signaling pathway in an LPS-induced RAW264.7 mouse macrophage inflammation model (41). Moreover, tanshinone IIA has been shown to suppress the TLR4/NF-κB signaling pathway in LPS-stimulated RAW264.7 macrophages (42) and to promote the phenotypic transition of macrophages from M1 to M2 (43). Similarly, cryptotanshinone inhibits the TLR4/NF-κB pathway (44) and facilitates M1 to M2 polarization (45). However, there is also report showing that cryptotanshinone can reprogram tumor-associated macrophages from the M2 to the M1 phenotype by regulating the TRAF6-ASK1 signaling axis (46), suggesting that SM and its bioactive compounds may act through distinct mechanisms within the tumor microenvironment. Overall, the regulatory mechanisms of this herb on macrophage polarization are diverse and complex, which may be due to variations in animal species, physiological status, disease models, as well as the composition and dosage of its bioactive compounds. Therefore, to further investigate these potential mechanisms, specifically in chickens, we used network pharmacology, molecular docking, and molecular dynamics simulation.

The NF-κB signaling pathway, a sub-branch of the TLR pathway, was identified as the core pathway in the network pharmacology analysis, while NFKBIA (which encodes IκBα) was recognized as the core target within this pathway. Furthermore, 15 pivotal bioactive compounds were identified from SM-compund target network. Molecular docking analysis revealed that among these 15 compounds, tanshinone IIA had the lowest binding energy to both the experimentally determined structure of NFKBIA and the structure predicted by AlphaFold 3. Additionally, molecular dynamics simulations confirmed that tanshinone IIA could bind stably to NFKBIA, strongly indicating that tanshinone IIA serves as the core compound in SM regulating chicken macrophage polarization. The NF-κB family subunits form homodimers or heterodimers in the cytoplasm, with the p50–p65 heterodimer representing the most predominant form. Under resting conditions, the p50–p65 heterodimer is sequestered in the cytoplasm by binding to IκBα (47). In the TLR4/NF-κB cascade, stimulation of TLR4 by ligands such as LPS triggers sequential signal transduction and activates the IκB kinase (IKK) complex. Activated IKK specifically phosphorylates two serine residues on IκBα. Subsequently, the E3 ubiquitin ligase SCF^β-TrCP^ mediates the conjugation of K48-linked ubiquitin chains to phosphorylated IκBα. Notably, IκBα can only expose its ubiquitination sites following phosphorylation. The 26S proteasome further recognizes K48 ubiquitin tags and degrades IκBα, thereby liberating the p50–p65 heterodimer. The released dimer then translocates into the nucleus and initiates the transcription of pro-inflammatory cytokines (48).

Accordingly, we hypothesized that the binding of tanshinone IIA to IκBα blocks IκBα phosphorylation and ubiquitination, thus prevents proteasomal degradation of IκBα, and ultimately restrains NF-κB nuclear translocation. Accumulated previous evidences support this hypothesis. For instance, tanshinone IIA inhibited LPS-induced IκBα phosphorylation and NF-κB nuclear translocation in RAW 264.7 macrophages in a dose-dependent manner (49). Similarly, tanshinone IIA markedly alleviated inflammatory damage by limiting IκBα degradation and NF-κB p65 phosphorylation in a mouse model of LPS-induced endometritis (50). Furthermore, tanshinone IIA treatment effectively suppressed IκB phosphorylation and NF-κB p65 nuclear entry, thereby inhibiting NF-κB cascade activation during RANKL-mediated osteoclast differentiation (51). Combining the *in vivo* findings and *in silico* bioinformatic analyses in the present study, we conclude that dietary SMUP facilitates M2 anti-inflammatory macrophage polarization by inhibiting the TLR4/NF-κB signaling pathway. The precise mechanism is that tanshinone IIA directly targets IκBα, prevents its phosphorylation and degradation, and subsequently reduces NF-κB nuclear translocation and pro-inflammatory gene expression.

The gut microbiota and barrier maintain a critical reciprocity, forming a key determinant of intestinal health and immune homeostasis (52). In the present study, Tenericutes and its subordinate taxa, including Mollicutes, Mycoplasmatales, and Mycoplasmataceae, were abnormally enriched in the CON group. Tenericutes contains a variety of pathogenic bacteria. For instance, Mycoplasmas can trigger intestinal inflammation and multiple diseases (53). Higher Tenericutes abundance has also been documented in the intestine with morphological damage and barrier dysfunction induced by enteral nutrition deprivation (54), heat stress (55), and mercury exposure (56). Moreover, hens with low laying performance exhibit a markedly higher relative abundance of intestinal Tenericutes compared with high-yield laying hens (57). Collectively, the excessive accumulation of Tenericutes in the chicken intestine may indicate impaired intestinal health and declined laying performance.

Functional analysis indicated that SMUP supplementation enhances the ansamycin biosynthesis activity of the gut microbiota. Ansamycins, a class of bacterial secondary metabolites that include rifaximin, exhibit antibacterial and anti-inflammatory activities and strengthen the intestinal epithelial barrier (58). Colucci et al. reported that rifaximin effectively reversed diclofenac-induced intestinal injury and inflammation by reducing ileal TNF-α and IL-1β levels, inhibiting the TLR-4/NF-κB pathway, upregulating *occludin* expression, and increasing beneficial *Lactobacillus* (59). In chickens, dietary magnolol attenuated the adverse effects of *Salmonella pullorum* and increased the pathways related to the biosynthesis of ansamycins (60). Therefore, dietary SMUP may protect the intestinal barrier and alleviate intestinal inflammation by enhancing microbial ansamycin biosynthesis in the gut. However, it should be noted that this result is a computational prediction from PICRUSt2 based on 16S rDNA sequencing data, rather than a direct measurement of metabolites or gene expression. Therefore, experimental validation—such as quantifying ansamycin level in the intestinal contents or examining the expression of key genes involved in ansamycin biosynthesis through gut microbial transcriptomics—is further required to confirm this prediction.

The network pharmacology, molecular docking, and molecular dynamics simulations findings suggested that tanshinone IIA targets NFKBIA (IκBα) and may therefore affect chicken macrophage polarization. Nevertheless, *in silico* methods have inherent limitations. For instance, the predicted compound–target interactions and binding modes require experimental validation using orthogonal approaches, such as surface plasmon resonance, cellular thermal shift assays, and functional studies (e.g., gene knockdown/overexpression or site-directed mutagenesis). Specifically, our computational analysis suggested that tanshinone IIA could inhibit NF-κB activation by blocking IκBα phosphorylation and ubiquitination—a hypothesis that must be directly tested by Western blot for IκBα phosphorylation and ubiquitination assays (e.g., co-immunoprecipitation coupled with ubiquitin blotting). Therefore, the *in silico* findings should be interpreted as hypothesis-generating rather than conclusive evidence, and rigorous experimental validation is necessary to fully decipher the mechanisms by which SMUP regulates intestinal immunity in chickens.

## 5 Conclusions

In summary, dietary SMUP (15 g/kg) effectively mitigated the decline in intestinal health of late-laying hens, as evidenced by improved ileal morphology, strengthened epithelial barrier, and reduced local inflammation. Moreover, dietary SMUP modulated the ileal microbiota by suppressing pathogenic Tenericutes and enriching predicted ansamycin biosynthesis. The increased plasma IL-10 and the reciprocal changes in M1-/M2-related gene expression suggest that dietary SMUP changes the intestinal macrophage polarization from the pro-inflammatory M1 toward the anti-inflammatory M2 phenotype. Mechanistically, integrated *in silico* analyses identified tanshinone IIA as a core compound directly targeting NFKBIA (IκBα) via tanshinone IIA, stabilizes IκBα, thereby blocking its phosphorylation-dependent degradation and subsequent NF-κB nuclear translocation. This action inhibits M1-type macrophage activation and promotes M2-biased responses. Collectively, dietary supplementation with 15 g/kg SMUP, as a valuable natural plant-derived feed additive, improves intestinal health identifies of hens during late laying period, and its bioactive compounds (such as tanshinone IIA) warrant in-depth further research.

## Data Availability

The 16S rDNA sequencing data presented in this study have been deposited in the NCBI Sequence Read Archive (SRA) under BioProject accession number PRJNA1512692. The processed ASV abundance table and KEGG level 3 pathway abundance table are available in the Figshare repository under DOI 10.6084/m9.figshare.33245991 (https://doi.org/10.6084/m9.figshare.33245991). Other raw data supporting the conclusions of this article will be made available by the authors, without undue reservation.
