# Supplementary material for: Salvia miltiorrhiza ultrafine powder improves ileal barrier function and health of hens during late laying period via TLR4/NF-κB-mediated macrophage polarization
Source: Front Immunol. 2026 Aug 20;17:1934610. doi: 10.3389/fimmu.2026.1934610 (PMC13538885; doi:10.3389/fimmu.2026.1934610)
Supplement: Supplementary file 1 [file Table1.docx]

**Table S1**

Composition and nutrient level of the basal diet (%; as dry matter basis).

| Ingredients | Content | Nutrient ^2^ | Level |
| --- | --- | --- | --- |
| Corn | 62.67 | Metabolic energy (MJ/kg) | 11.25 |
| Soybean meal | 23.88 | Crude protein | 14.23 |
| Limestone | 7.96 | Ether extract | 9.66 |
| Soybean oil | 0.49 | Calcium | 3.51 |
| Premix ^1^ | 5.00 | Available phosphorus | 0.34 |
| Total | 100.00 | Lysine | 0.71 |
|  |  | Methionine | 0.37 |

^1^ The premix provided the following per kg of diets: vitamin A, 140,000 IU; vitamin D_3_, 50,000 IU; vitamin E, 480 mg; vitamin Κ, 3,180 mg; vitamin B_1_, 63 mg; vitamin B_2_, 200 mg; vitamin B_6_, 140 mg; vitamin B_12_, 0.7 mg; nicotinic acid, 1000 mg; *D*-pantothenic acid, 500 mg; folic acid, 50 mg; *D*-biotin, 5.0 mg; choline chloride, 900 mg; Fe (as ferrous sulfate), 2.0 g; Cu (as copper sulfate), 0.3 g; Mn (as manganese sulfate), 1.8 g; Zn (as zinc sulfate), 2.0 g; I (as potassium iodide), 70 mg; Se (as sodium selenite), 9 mg; methionine, 1 g; phytase, 3,000 IU.

^2^ Crude protein and ether extract are measured values, and the others are calculated value.

**Table S2**

Primers used for quantitative real-time PCR.

| Gene^1^ | Accession No. | Primer sequence (5′→3′) | Product size (bp) |
| --- | --- | --- | --- |
| *Claudin-1* | NM_001013611.2 | F: GGGTCTGGTTGGTGTGTTTG  R: TCTGGTGTTAACGGGTGTGA | 205 |
| *Claudin-5* | NM_204201.2 | F: CATCACTTCTCCTTCGTCAGC  R: GCACAAAGATCTCCCAGGTC | 111 |
| *Occludin* | XM_046904540.1 | F: ACAGCCCTCAATACCAGGATGTG  R: ACCATGCGCTTGATGTGGAA | 133 |
| *ZO-1* | XM_046925214.1 | F: TATAGAAGATCGTGCGCCTCC  R: GAGGTCTGCCATCGTAGCTC | 209 |
| *MUC2* | XM_040673077.2 | F: GCTACAGGATCTGCCTTTGC  R: AATGGGCCCTCTGAGTTTTT | 152 |
| *TLR4* | NM_001030693.2 | F: CCACTATTCGGTTGGTGGAC  R: ACAGCTTCTCAGCAGGCAAT | 120 |
| *TLR5* | XM_046914374.1 | F: CCTTGTGCTTTGAGGAACGAGA  R: CACCCATCTTTGAGAAACTGCC | 124 |
| *MyD88* | NM_001030962.5 | F: GATGATCCGTATGGGCATGGA  R: ATGGACCACACACACGTTCC | 170 |
| *NF-κB p65* | XM_046915553.1 | F: TGCCTTTTGCTTGAGGGTGATG  R: CTGCCAGTTTTGTGAAGCCC | 100 |
| *IL-1β* | NM_204524.2 | F: ACTGGGCATCAAGGGCTA  R: GGTAGAAGATGAAGCGGGTC | 131 |
| *IL-6* | NM_204628.2 | F: AATCCCTCCTCGCCAATCTG  R: GCCCTCACGGTCTTCTCCATA | 106 |
| *TNF-α* | NM_204267.2 | F: CCGTAGTGCTGTTCTATGACCG  R: GTTCCACATCTTTCAGAGCATCAA | 189 |
| *IFN-γ* | NM_205149.2 | F: AAAGCCGCACATCAAACACA  R: GCCATCAGGAAGGTTGTTTTTC | 64 |
| *iNOS* | NM_204961.2 | F: CAGCTGATTGGGTGTGGAT  R: TTTCTTTGGCCTACGGGTC | 158 |
| *IL-4* | NM_001007079.2 | F: GTGCCCACGCTGTGCTTAC  R: AGGAAACCTCTCCCTGGATGTC | 82 |
| *IL-10* | NM_001004414.4 | F: CGCTGTCACCGCTTCTTCA  R: TCCCGTTCTCATCCATCTTCTC | 88 |
| *IL-13* | NM_001195791.2 | F: AGTGCTGGACAACATGACCGA  R: GCAAGAAGTTCCGCAGGTAGAT | 128 |
| *β-actin* | NM_205518.2 | F: AGTACCCCATTGAACACGGT  R: ATACATGGCTGGGGTGTTGA | 197 |

^1^*ZO*, zonula occludens; *MUC2*, mucin-2; *TLR*, toll-like receptor; *MyD88*, myeloid differentiation factor 88; *NF-κB*, nuclear factor kappa; *IL*, interleukin; *TNF-α*, tumor necrosis factor α; *IFN-γ*, interferon-γ; *iNOS*, inducible nitric oxide synthase.

**Table S3**

Information of potential bioactive compounds of *Salvia miltiorrhiza.*

| Mol ID | No. | Molecule name | OB (%) ^1^ | DL ^2^ |
| --- | --- | --- | --- | --- |
| MOL007132 | SM1 | (2R)-3-(3,4-dihydroxyphenyl)-2-[(Z)-3-(3,4-dihydroxyphenyl)acryloyl]oxy-propionic acid | 109.38 | 0.35 |
| MOL007155 | SM2 | (6S)-6-(hydroxymethyl)-1,6-dimethyl-8,9-dihydro-7H-naphtho[8,7-g]benzofuran-10,11-dione | 65.26 | 0.45 |
| MOL007150 | SM3 | (6S)-6-hydroxy-1-methyl-6-methylol-8,9-dihydro-7H-naphtho[8,7-g]benzofuran-10,11-quinone | 75.39 | 0.46 |
| MOL007070 | SM4 | (6S,7R)-6,7-dihydroxy-1,6-dimethyl-8,9-dihydro-7H-naphtho[8,7-g]benzofuran-10,11-dione | 41.31 | 0.45 |
| MOL007048 | SM5 | (E)-3-[2-(3,4-dihydroxyphenyl)-7-hydroxy-benzofuran-4-yl]acrylic acid | 48.24 | 0.31 |
| MOL007140 | SM6 | (Z)-3-[2-[(E)-2-(3,4-dihydroxyphenyl)vinyl]-3,4-dihydroxy-phenyl]acrylic acid | 88.54 | 0.26 |
| MOL001601 | SM7 | 1,2,5,6-tetrahydrotanshinone | 38.75 | 0.36 |
| MOL007127 | SM8 | 1-methyl-8,9-dihydro-7H-naphtho[5,6-g]benzofuran-6,10,11-trione | 34.72 | 0.37 |
| MOL007050 | SM9 | 2-(4-hydroxy-3-methoxyphenyl)-5-(3-hydroxypropyl)-7-methoxy-3-benzofurancarboxaldehyde | 62.78 | 0.40 |
| MOL007041 | SM10 | 2-isopropyl-8-methylphenanthrene-3,4-dione | 40.86 | 0.23 |
| MOL007059 | SM11 | 3-beta-hydroxymethyllenetanshiquinone | 32.16 | 0.41 |
| MOL007045 | SM12 | 3α-hydroxytanshinone Ⅱ A | 44.93 | 0.44 |
| MOL007049 | SM13 | 4-methylenemiltirone | 34.35 | 0.23 |
| MOL007036 | SM14 | 5,6-dihydroxy-7-isopropyl-1,1-dimethyl-2,3-dihydrophenanthren-4-one | 33.77 | 0.29 |
| MOL002776 | SM15 | Baicalin | 40.12 | 0.75 |
| MOL007107 | SM16 | Ferruginol | 36.07 | 0.25 |
| MOL007088 | SM17 | Cryptotanshinone | 52.34 | 0.40 |
| MOL007082 | SM18 | Danshenol A | 56.97 | 0.52 |
| MOL007081 | SM19 | Danshenol B | 57.95 | 0.56 |
| MOL007094 | SM20 | Danshenspiroketallactone | 50.43 | 0.31 |
| MOL007134 | SM21 | Danshensu | 36.91 | 0.06 |
| MOL007093 | SM22 | Dan-shexinkum D | 38.88 | 0.55 |
| MOL002651 | SM23 | Dehydrotanshinone II A | 43.76 | 0.40 |
| MOL007098 | SM24 | Deoxyneocryptotanshinone | 49.40 | 0.29 |
| MOL000569 | SM25 | Digallate | 61.85 | 0.26 |
| MOL007100 | SM26 | Dihydrotanshinlactone | 38.68 | 0.32 |
| MOL007101 | SM27 | Dihydrotanshinone I | 45.04 | 0.36 |
| MOL007105 | SM28 | Epidanshenspiroketallactone | 68.27 | 0.31 |
| MOL007058 | SM29 | Formyltanshinone | 73.44 | 0.42 |
| MOL007108 | SM30 | Isocryptotanshinone | 54.98 | 0.39 |
| MOL001942 | SM31 | Isoimperatorin | 45.46 | 0.23 |
| MOL007111 | SM32 | Isotanshinone II | 49.92 | 0.40 |
| MOL007113 | SM33 | Lithospermic acid | 2.67 | 0.76 |
| MOL000006 | SM34 | Luteolin | 36.16 | 0.25 |
| MOL007115 | SM35 | Manool | 45.04 | 0.20 |
| MOL007061 | SM36 | Methylenetanshinquinone | 37.07 | 0.36 |
| MOL007118 | SM37 | Microstegiol | 39.61 | 0.28 |
| MOL007119 | SM38 | Miltionone I | 49.68 | 0.32 |
| MOL007120 | SM39 | Miltionone Ⅱ | 71.03 | 0.44 |
| MOL007121 | SM40 | Miltipolone | 36.56 | 0.37 |
| MOL007122 | SM41 | Miltirone | 38.76 | 0.25 |
| MOL007125 | SM42 | Neocryptotanshinone | 52.49 | 0.32 |
| MOL007124 | SM43 | Neocryptotanshinone II | 39.46 | 0.23 |
| MOL001771 | SM44 | Poriferast-5-en-3beta-ol | 36.91 | 0.75 |
| MOL001659 | SM45 | Poriferasterol | 43.83 | 0.76 |
| MOL007130 | SM46 | Prolithospermic acid | 64.37 | 0.31 |
| MOL001452 | SM47 | Protocatechualdehyde | 38.35 | 0.03 |
| MOL007063 | SM48 | Przewalskin A | 37.11 | 0.65 |
| MOL007064 | SM49 | Przewalskin B | 110.32 | 0.44 |
| MOL007068 | SM50 | Przewaquinone B | 62.24 | 0.41 |
| MOL007069 | SM51 | Przewaquinone C | 55.74 | 0.40 |
| MOL007152 | SM52 | Przewaquinone E | 42.85 | 0.45 |
| MOL007071 | SM53 | Przewaquinone F | 40.31 | 0.46 |
| MOL011865 | SM54 | Rosmarinic acid | 1.38 | 0.35 |
| MOL007136 | SM55 | Salvianolic acid A | 2.96 | 0.70 |
| MOL007074 | SM56 | Salvianolic acid B | 3.01 | 0.41 |
| MOL007141 | SM57 | Salvianolic acid G | 45.56 | 0.61 |
| MOL007142 | SM58 | Salvianolic acid J | 43.38 | 0.72 |
| MOL007085 | SM59 | Salvilenone | 30.38 | 0.38 |
| MOL007143 | SM60 | Salvilenone I | 32.43 | 0.23 |
| MOL007145 | SM61 | Salviolone | 31.72 | 0.24 |
| MOL007077 | SM62 | Sclareol | 43.67 | 0.21 |
| MOL002222 | SM63 | Sugiol | 36.11 | 0.28 |
| MOL007079 | SM64 | Tanshinaldehyde | 52.47 | 0.45 |
| MOL007151 | SM65 | Tanshindiol B | 42.67 | 0.45 |
| MOL007156 | SM66 | Tanshinone VI | 45.64 | 0.30 |
| MOL007157 | SM67 | Tanshinone I | 29.27 | 0.36 |
| MOL007154 | SM68 | Tanshinone IIA | 49.89 | 0.40 |
| MOL006824 | SM69 | α-amyrin | 39.51 | 0.76 |

^1^ OB: oral bioavailability

^2^ DL: drug likeness

**Table S4**

Potential targets of *Salvia miltiorrhiza* compounds acting on chicken macrophage polarization.

| Gene name | Full name |
| --- | --- |
| ABCA1 | ATP-binding cassette subfamily A member 1 |
| ACTA2 | Actin, aortic smooth muscle |
| ADIPOQ | Adiponectin |
| CCL5 | C-C motif chemokine 5 |
| CCR2 | C-C motif chemokine receptor 2 |
| CCR5 | C-C chemokine receptor type 5 |
| CD83 | B-cell activation protein |
| CD86 | T-lymphocyte activation antigen CD86 |
| CEBPB | CCAAT/enhancer-binding protein β |
| CSF1R | Macrophage colony-stimulating factor 1 receptor |
| CSF3 | Granulocyte colony-stimulating factor |
| CXCR1 | C-X-C chemokine receptor type 1 |
| FTH1 | Ferritin heavy chain 1 |
| HCK | Hematopoietic cell kinase |
| HRAS | HRas proto-oncogene GTPase |
| IL1B | Interleukin-1 β |
| JAK3 | Janus kinase 3 |
| JUN | Transcription factor Jun |
| LYZ | Lysozyme C |
| MAP3K8 | Mitogen-activated protein kinase kinase kinase 8 |
| MME | Membrane metalloendopeptidase |
| MMP1 | Matrix metalloproteinase-1 |
| MMP2 | Matrix metalloproteinase-2 |
| NFKBIA | NF-κ-B inhibitor α |
| NOS2 | Nitric oxide synthase 2 |
| PDGFRB | Platelet-derived growth factor receptor β |
| PRKCH | Protein kinase C eta type |
| PTGS2 | Prostaglandin-endoperoxide synthase 2 |
| PTPN2 | Protein tyrosine phosphatase non-receptor type 2 |
| RXRA | Retinoid X receptor α |
| TBXAS1 | Thromboxane A synthase 1 |
| XDH | Xanthine dehydrogenase |

**Table S5**

Top 10 pathways (ranked by *P*-value) in KEGG enrichment analysis of 32 potential targets for *Salvia miltiorrhiza* compounds acting on chicken macrophage polarization.

| Pathway | Genes^1^ | *P*-values | Gene ratio (%)^2^ |
| --- | --- | --- | --- |
| Toll-like receptor signaling pathway | NFKBIA, CD86, JUN, IL1B, CCL5, MAP3K8 | 0.0000315 | 18.75 |
| Cytokine-cytokine receptor interaction | CSF1R, CSF3, CXCR1, IL1B, CCL5, CCR5, CCR2 | 0.000198 | 21.88 |
| C-type lectin receptor signaling pathway | NFKBIA, JUN, IL1B, PTGS2, HRAS | 0.000532 | 15.63 |
| MAPK signaling pathway | PDGFRB, CSF1R, JUN, IL1B, MAP3K8, HRAS | 0.00477 | 18.75 |
| NOD-like receptor signaling pathway | NFKBIA, JUN, IL1B, CCL5 | 0.0188 | 12.50 |
| Virion-Human immunodeficiency virus | CCR5, CCR2 | 0.022 | 6.25 |
| Cytosolic DNA-sensing pathway | NFKBIA, IL1B, CCL5 | 0.0236 | 9.38 |
| Efferocytosis | ABCA1, CEBPB, RXRA, PTGS2 | 0.0256 | 12.50 |
| PPAR signaling pathway | RXRA, MMP1, ADIPOQ | 0.0314 | 9.38 |
| Adipocytokine signaling pathway | NFKBIA, RXRA, ADIPOQ | 0.0324 | 9.38 |

^1^Genes enriched in the KEGG pathway from the 32 potential targets.

^2^Gene Ratio (%) = (Number of genes enriched in the KEGG pathway from the 32 potential targets)/32×100.
